# Forecasting influenza outbreak dynamics in Melbourne from Internet search query surveillance data

**DOI:** 10.1111/irv.12376

**Published:** 2016-03-07

**Authors:** Robert Moss, Alexander Zarebski, Peter Dawson, James M. McCaw

**Affiliations:** ^1^ Centre for Epidemiology and BiostatisticsMelbourne School of Population and Global HealthThe University of MelbourneMelbourneAustralia; ^2^ School of Mathematics and StatisticsThe University of MelbourneMelbourneAustralia; ^3^ Land Personnel Protection BranchLand DivisionDefence Science and Technology GroupMelbourneAustralia; ^4^ Modelling & SimulationMurdoch Childrens Research InstituteRoyal Childrens HospitalMelbourneAustralia

**Keywords:** Bayesian prediction, epidemic forecasting, influenza

## Abstract

**Background:**

Accurate forecasting of seasonal influenza epidemics is of great concern to healthcare providers in temperate climates, as these epidemics vary substantially in their size, timing and duration from year to year, making it a challenge to deliver timely and proportionate responses. Previous studies have shown that Bayesian estimation techniques can accurately predict when an influenza epidemic will peak many weeks in advance, using existing surveillance data, but these methods must be tailored both to the target population and to the surveillance system.

**Objectives:**

Our aim was to evaluate whether forecasts of similar accuracy could be obtained for metropolitan Melbourne (Australia).

**Methods:**

We used the bootstrap particle filter and a mechanistic infection model to generate epidemic forecasts for metropolitan Melbourne (Australia) from weekly Internet search query surveillance data reported by Google Flu Trends for 2006–14.

**Results and Conclusions:**

Optimal observation models were selected from hundreds of candidates using a novel approach that treats forecasts akin to receiver operating characteristic (ROC) curves. We show that the timing of the epidemic peak can be accurately predicted 4–6 weeks in advance, but that the magnitude of the epidemic peak and the overall burden are much harder to predict. We then discuss how the infection and observation models and the filtering process may be refined to improve forecast robustness, thereby improving the utility of these methods for healthcare decision support.

## Introduction

Outside of the tropics, influenza viruses produce substantial burden on healthcare systems in a highly seasonal manner, with high prevalence in the winter months and very low prevalence in the summer months. Despite the regularity with which seasonal influenza outbreaks occur, there is substantial variation in the outbreak timing (onset and peak) and outbreak size (cumulative and peak burden), across different cities and countries in the same season, and also for the same city or country from season to season.

For this reason, the ability to predict the progression of an epidemic well in advance is of great utility for enabling healthcare providers to make best use of the resources at their disposal. A variety of recursive Bayesian estimation methods (‘filters’) have been used for such forecasting purposes,^1^, ^2^ often in combination with Internet search query surveillance data and mechanistic models of infection.^3^, ^4^, ^5^, ^6^, ^7^, ^8^

While it has been shown that these methods may be of practical forecasting use in future influenza seasons, they must be adapted to the vagaries of both the surveillance system from which the data (‘observations’) are collected and the population being surveilled. Here, we demonstrate that accurate forecasts of influenza epidemics in metropolitan Melbourne (Australia) can be obtained from the combination of a particle filter, an SEIR model of influenza transmission, an observation model based upon a negative binomial distribution and influenza‐like illness (ILI) data obtained from Google Flu Trends.^9^ While Google is no longer publishing estimates of disease activity (as of August 20, 2015), they continue to provide signal data for research purposes.^10^ The methods and results presented here are the first step towards a decision‐support tool that we will use to generate near‐real‐time forecasts of the Melbourne influenza season in 2015 and beyond.

## Methods

A recent comparison of filtering methods for influenza epidemic forecasting (applied to 115 cities in USA) found that the peak timing forecasts were comparably accurate for the six surveyed methods.^11^ It was also observed that the particle filters performed ‘slightly better predicting peaks 1–5 weeks in the future’, while ‘ensemble [Kalman] filters were better at indicating that the seasonal peak had already occurred’.

**Figure 1:**
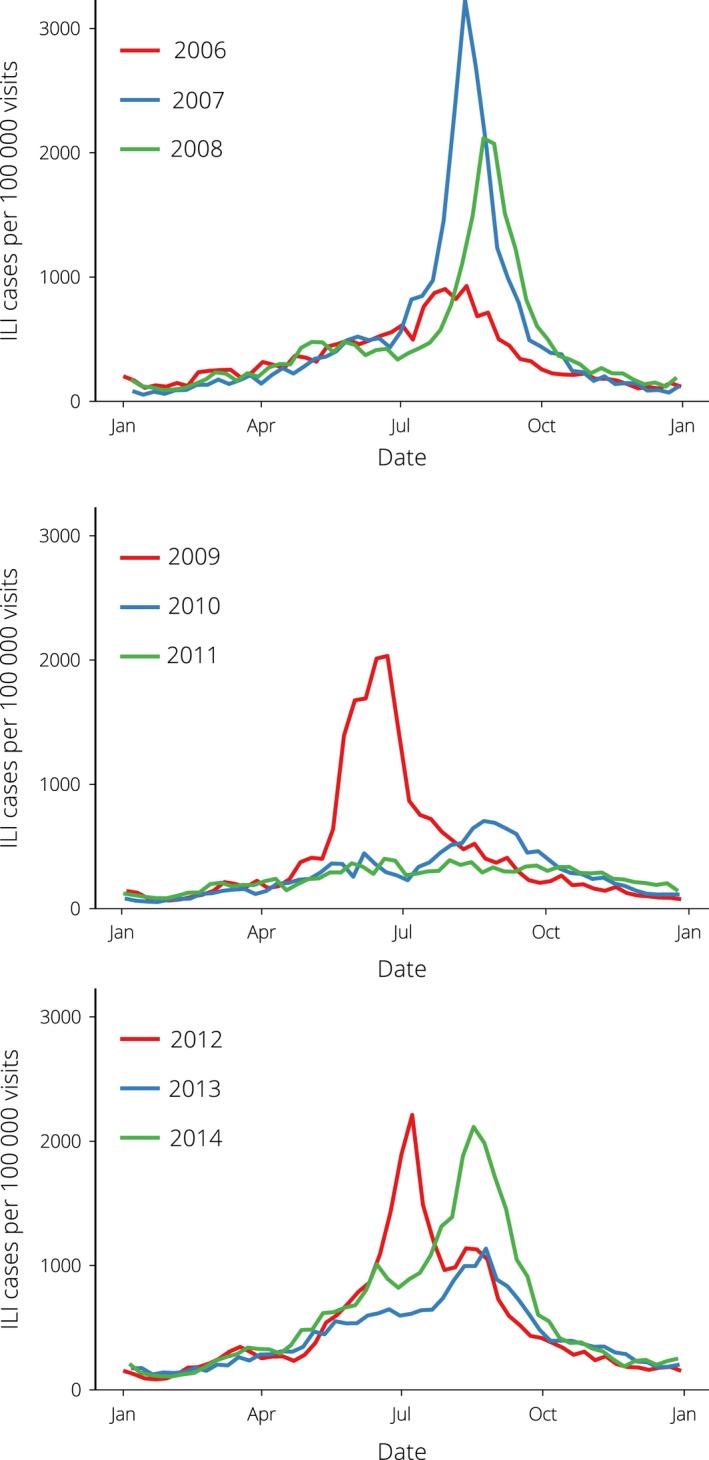
Google Flu Trends data for Victoria.

We applied the bootstrap particle filter method to determine which realisations of an SEIR compartment model were the most likely to yield *in silico* observations consistent with the weekly ILI data reported by Google Flu Trends for the state of Victoria, for each of the available calendar years (2006–2014, shown in Figure 1). We assumed that the data characterised influenza activity in metropolitan Melbourne, which comprises just over 75% of the Victorian population and has higher Internet penetration than the rest of the state. In this approach, a finite set of model vectors (particles) are used to approximate the continuous model‐space likelihood distribution. As real‐world observations are obtained, the particle likelihoods (weights) are updated and, when the likelihood is too highly concentrated in too few of the particles, the particles are *resampled* to distribute the likelihood more uniformly.

Each particle encompasses the state variables and model parameters for an SEIR compartment model, whose initial values are drawn from prior distributions (see Supplementary Material section S1 for details). Stochastic noise is included in the flows between compartments and in the model parameters, as per Skvortsov and Ristic.^6^ The entire population is assumed to be susceptible at the start of the calendar year, and an initial exposure occurs with daily probability *p*_*seed*_.

While a fraction of the Melbourne population will be immune to the circulating strain(s), the use of a wholly susceptible model population is not problematic as we are not attempting to infer properties of the true population. Were we to impose some prior immunity (*0 < R(0) < N*) the *effective* reproduction number (*R*_*Eff*_) would be unchanged, but the filter would converge on particles with larger values for *R*_*0,*_ 12, 13 and the likelihood of observing an infected individual (which relates model incidence to the observed data) would only need to be scaled by some constant.

Particles are initially assigned uniform weights, which are subsequently adjusted in response to each observation and normalised so as to sum to one (see Supplementary Material section S2 for details). When the *effective* number of particles drops below the threshold *N*_*min*_, the particles are *resampled* in proportion to their weights; this is done using the systematic (deterministic) method, as described by Kitagawa.^14^

The observation model (see Supplementary Material section S3 for details) relates disease incidence in the SEIR model to rates of ILI presentation, based on 2012 population statistics obtained from Victorian Department of Health & Human Services (DHHS) reports for the metropolitan Melbourne regions (Eastern, North & West, Southern).^15^

Google Flu Trends reports ILI prevalence as *integer counts* of ILI cases per 100,000 GP visits, and so we can express the daily probability of an individual presenting with ILI in the absence of influenza as a function of the (imposed) background ILI rate *B*_*R*_ and the daily number of GP visits per individual (*v*_*daily*_). The value for *v*_*daily*_ was obtained from the reported annual rate of 5,615 GP attendances per 1,000 population.^15^ The background ILI rate accounts for both out‐of‐season influenza importations (which do not lead to ongoing community transmission) and incidence of other respiratory infections. This rate was *not* subtracted from the weekly ILI estimates, to avoid the introduction of a systematic bias.

The probability that an infected individual will visit a GP and be identified as having ILI (*p*_*id*_) is a parameter of the observation model. The probability that an individual will be identified as an ILI case is the sum of two independent events: becoming infectious and subsequently being identified (*p*_*id*_), or not becoming infectious but presenting with an ILI. The probability of becoming infectious is defined as the fraction of the population that became infectious (i.e. transitioned from state *E* to state *I*) during the time interval, and subsumes both symptomatic and asymptomatic infections. In the absence of reliable data to the contrary, both types of infection are assumed to be identically infectious. The observation probability (*p*_*id*_) therefore represents the probability of an infection being symptomatic *and* observed.

**Figure 2:**
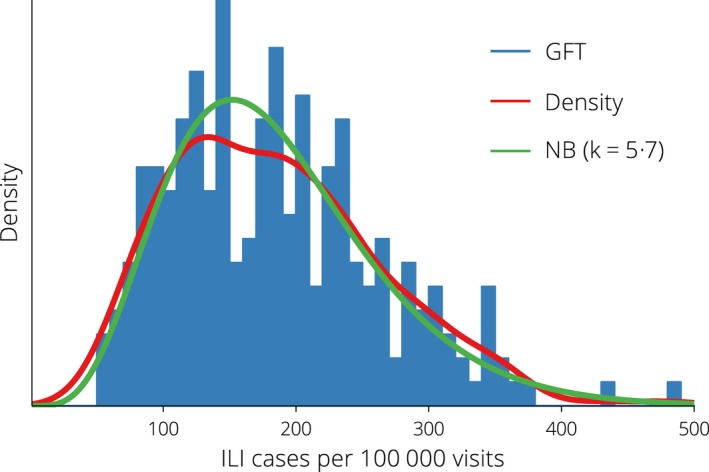
Distribution of the Google Flu Trends data outside of the influenza season (i.e, from November to April, inclusive), accompanied by a kernel density estimate and a maximum‐likelihood negative binomial distribution with parameters k = 5.7 (95% CI: 5.61, 5.75) and E[y_k_] = 184.9 (95% CI: 184.3, 185.6).

The available surveillance data comprise non‐negative integer counts for which the variance is often as large as the mean; they are overdispersed with respect to a Poisson distribution. A variety of distributions are suitable for modelling overdispersed count data,^16^ but differ in their mean–variance relationships. The surveillance data exhibit an (approximately) quadratic mean–variance relationship, which suggests that a negative binomial distribution is an appropriate choice^17^ to define the likelihood of obtaining an ILI presentation rate from a given particle. This distribution is frequently used in ecology^18^, ^19^ and epidemiology^20^, ^21^, ^22^ because it allows the mean and variance to be controlled independently,^16^ and it provides a good *phenomenological* description of count data^23^ (see Figure 2). The *dispersion parameter k* controls the variance: as *k* increases the variance decreases and the distribution approaches the Poisson.

To account for the variation in peak incidence and outbreak duration, we systematically varied the background ILI rate (*B*_*R*_ *∈ {200,250,300,350*}), the dispersion parameter (*k ∈ {1,10,100,1000*}) and the probability of ILI observation per infection (*p*_*id*_ ∈ *{0·0025, 0·0050,…, 0·1000}*). For each combination of these observation model parameters, we generated forecasts for every calendar year (2006–2014) after every observation from mid‐March onwards. For each forecast, we calculated the estimated ILI incidence over the past week at *every day* starting from the forecasting date (i.e. the date of the most recent observation) until the end of the year (i.e. the week ending 31 December). In this way, each forecast comprised estimates of weekly ILI incidence for every 7‐day period from the forecasting date until the end of the year, and peak incidence could occur in a week ending on any weekday. In order to determine forecast accuracy with respect to the original data (reported for weeks ending on Sundays), we used a tolerance of ± 10 days, equivalent to rounding the peak to the nearest Sunday (i.e. ±3 days) and defining accuracy to mean ± 1 week. By summing the accuracy over the 8 weeks prior to the true peak – similar in concept to calculating the area under a ROC curve – we obtained annual ‘scores’ for each observation model. Observations models were then ranked by their mean annual scores.

## Results

Google Flu Trends provides weekly ILI estimates for the 2006–2014 calendar years for the state of Victoria (assumed to be representative of ILI levels in metropolitan Melbourne), as shown in Figure 1. Peak incidence rates vary from 390 cases to 3,229 cases per 100 000 GP visits (cf. 2011 and 2007), and the peak timing varies between mid‐June and late August. Optimal forecasting results were obtained for *B*_*R*_ = 300_,_ *k* = 10, and 0·004 ≤ *p*_*id*_. ≤ 0·006. Here, we present the forecasting results obtained using *B*_*R*_ = 300 and *p*_*id*_ = 0·05, which are representative of the results obtained across the optimal range of values for *p*_*id*_. Forecasts for every calendar year using all combinations of the above parameters are included in the supplementary material.

**Figure 3:**
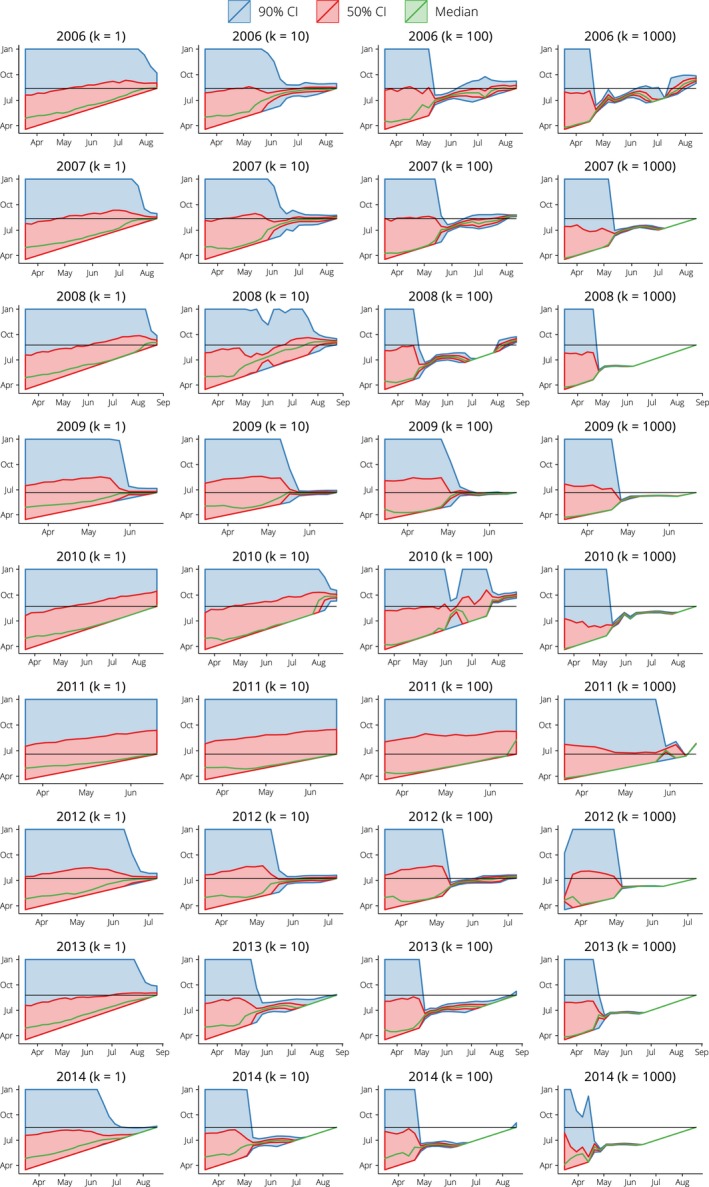
Predicted timing of the epidemic peaks plotted by forecasting date (until the true peak is reached), for B_R_ = 300, p_id_ = 0.05 and several values of the dispersion parameter k. The black horizontal lines show the true timing. Note that the scale of the x‐axis differs between calendar years, since it extends from mid‐March until the time of the true peak.

The predicted timing of the epidemic peak for each year is shown in Figure 3 for each value of the dispersion parameter *k*, where the *x*‐axis indicates the date at which each forecast was generated. The effect of the dispersion parameter is evident in the width of the forecast credible intervals: when *k = 1*_,_ the likelihood distribution is overly wide and the forecasting confidence is necessarily low; when *k = 1000*_,_ the likelihood distribution is very narrow and particle degeneracy is frequently observed (e.g. 2007, 2008, 2013, 2014). The most accurate and precise forecasts are obtained when *k = 10* and *k = 100*, with particle degeneracy occurring less often for *k = 10* (e.g. 2008).

**Figure 4:**
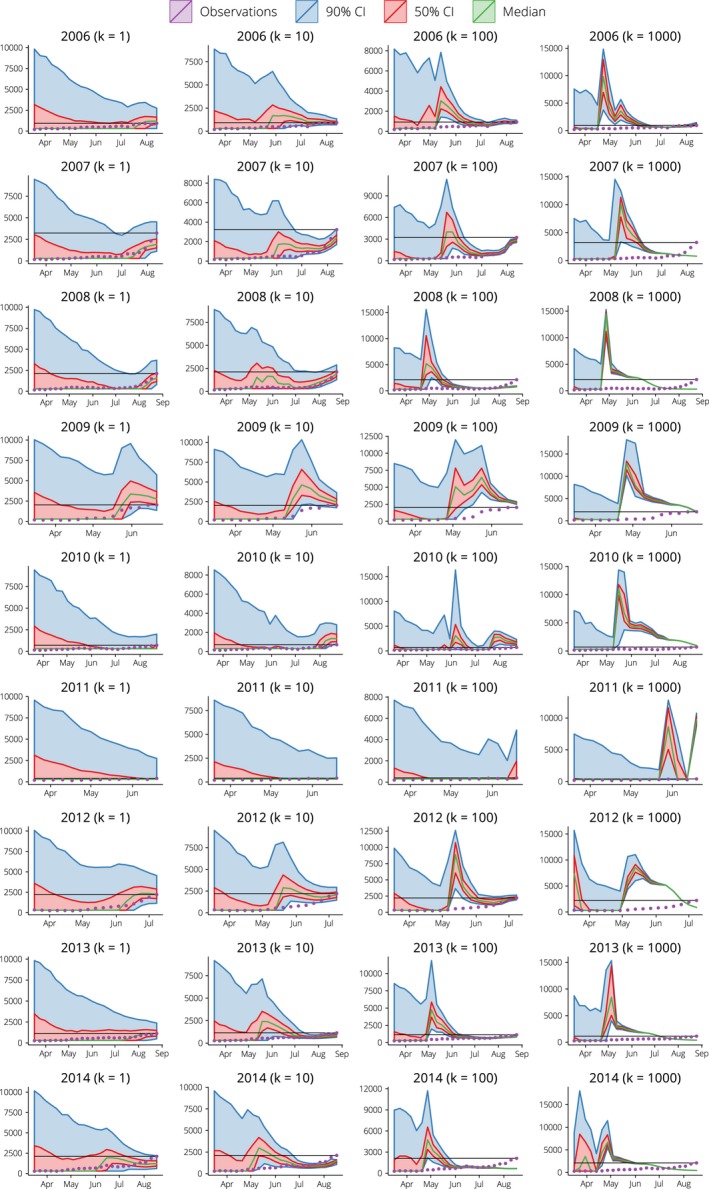
Predicted size of the epidemic peaks plotted by forecasting date (until the true peak is reached) and shown against the Google Flu Trends data, for B_R_ = 300, p_id_ = 0.05 and several values of the dispersion parameter k. The black horizontal lines show the true size of the peak. Note that the scale of the x‐axis differs between calendar years, since it extends from mid‐March until the time of the true peak.

The predicted size of the epidemic peak for each year is shown in Figure 4. In contrast to the peak timing forecasts, the peak size forecasts are both less precise (i.e. wider credible intervals) and less accurate. This comes as no surprise; when a nascent epidemic exhibits exponential growth in prevalence, the size of the resulting epidemic peak is much more sensitive to the precise growth rate than the timing of the peak (as measured from the early‐growth phase). It can also be seen that the value of the dispersion parameter *k* has similar effects on the peak size forecasts as on the peak timing forecasts. When *k ≥ 100*_,_ particle degeneracy frequently occurs (e.g. 2007, 2008, 2012) and when *k = 1* the credible intervals are always wide. When *k = 10*, the particle filter is sometimes able to predict the peak size both accurately (± 33%) and precisely, from one or 2 weeks in advance of the true peak (2008, 2013) to up to 5 weeks in advance (2006, 2012).

Both the peak timing and peak size forecasts exhibit substantial variations from year to year, with the least accurate fits obtained when there is no clear epidemic peak (2010, 2011) or when an earlier, smaller peak is present (2014). The forecasts for 2014 might have been improved by smoothing the peak in June 2014 for all forecasts generated after June, once it became apparent that it was not the main epidemic peak, but smoothing the incoming surveillance data is beyond the scope of the study presented here.

For the remaining calendar years (2006–2009, 2012, 2013), it is clear that, given appropriate observation model parameters, the particle filter can steadily refine the forecasts and narrow in on the epidemic curve. As shown in Figure S1, the variance of both the peak size and timing forecasts decreases substantially as the forecasting date approaches the date of the true epidemic peak. Forecast accuracy increases as the forecast variance decreases (Figure S2) and so the accuracy increases as the true peak is approached (Figure S3). For example, when using a threshold of ± 10 days as the definition of an ‘accurate’ prediction of the peak timing (equivalent to ± 1 week, when aggregating dates into weekly bins), it can be seen that in 4 of the calendar years under consideration, more than 50% of the weighted forecasts accurately predicted the timing of the peak 5 weeks in advance.

## Discussion

### Principal findings

For flu seasons where there was a clear epidemic peak, the timing of this peak was accurately predicted 4–6 weeks in advance (±10 days); prediction of the peak size was substantially more difficult and was only ever achieved on an inconsistent basis 1–5 weeks prior to the peak (±33% of true size).

### Study strengths and weaknesses

In this study, we have robustly explored the relationship between forecasting accuracy and observation model (i.e. the case ascertainment proportion and overdispersion). The most accurate forecasts were obtained with a dispersion parameter of 10 (overdispersed with respect to the Poisson) and an ascertainment proportion of 5%. Note that we do not assume that parameters or variables are distributed according to any specific distribution; this flexibility is a feature of particle filter methods and allows the model posteriors to better approximate their true distributions when these distributions are, for example skewed, overdispersed or multimodal. The penalty is an increase in the computational cost of the forecasting algorithm due to the number of particles required to generate the ensemble forecasts. Our implementation is written in Python and can generate weekly forecasts from mid‐March until the epidemic peak (as shown in Figures 3 and 4) in 20–30 minutes on a standard desktop computer.

Google Flu Trends is a ‘black box’ whose internals are updated at unknown intervals, so the precise relationship between the data and ILI incidence in the target population remains uncertain.^24^, ^25^ As the algorithm is based on Internet searches,^9^ differences in Internet penetration and/or utilisation between populations presumably influence the quality of the data,^26^ which highlights the importance of evaluating these forecasting techniques in an Australian context. Moreover, this lack of transparency renders it difficult to decide when and how the observation model should be adjusted to account for changes to the Google Flu Trends algorithm. In recognition of these limitations, Google no longer publishes estimates of disease activity, but continues to provide signal data to researchers.^10^

For particular calendar years (e.g. 2008 and 2014 for *k = 10*) and choice of observation model, particle degeneracy was observed, indicating that the observation model was inaccurate or that the infection model priors were too narrow. When producing real‐time forecasts in the nascent stages of an ongoing (‘live’) influenza epidemic, these forecasts should be discarded and the particle filter restarted with an adjusted observation model and/or expanded infection model priors. As the epidemiological parameters of seasonal influenza strains are relatively well quantified and the infection model priors are already quite broad, the observation model parameters should be adjusted first. Where the effective number of particles decreases substantially in response to a single observation, it might be prudent to decrease the dispersion parameter and recalculate the particle weights. We explored a number of approaches to improve forecast robustness, including the use of Gaussian observation models, increasing the number of particles, and lowering the resampling threshold, but particle degeneracy was only avoided in the problematic seasons by compromising the forecasting performance in the remaining seasons (see Supplementary Material section S4 for details).

In addition to the peak size being a more difficult quantity to predict than the timing of the peak, it is also sensitive to our assumed probability of ILI observation per infection (*P*_*id*_), which acts as a linear scaling factor between model incidence and the surveillance data. Our chosen range of values for this parameter may have adversely affected the forecasts for 2007 (where the peak was 50% higher than in any other year) and, perhaps, for 2011 (where no peak was evident). Note that ‘2007 saw the most severe influenza season [in Australia] since national reporting of influenza began in 2001’,^27^ and Victorian isolates were mainly type A(H3N2),^27^ which is typically associated with more severe flu seasons. In contrast, the 2011 season was relatively mild^28^, ^29^ with a substantially higher proportion of influenza B notifications than in previous years.^29^

### Comparison with other studies

Several studies in recent years have combined recursive Bayesian estimation methods with mechanistic models of disease transmission for the purposes of early outbreak detection, epidemic forecasting and pathogen characterisation. With regard to seasonal influenza, existing forecasting studies have focused on cities across the USA, but as Yang *et al*. state, ‘the performance of any filter may be application dependent’.^11^ The results presented here are the first application of such forecasting methods to Australian data and demonstrate that we are able to obtain similar forecasting accuracy in the Australian context.

Shaman *et al*.^4^, ^5^ used an ensemble adjustment Kalman filter (EAKF) and an SIRS infection model to predict seasonal influenza outbreaks in 108 cities across the USA and showed that outbreak peak timing could be predicted 4–6 weeks in advance. They also found that predictions that the 2012–13 peak had passed were frequently incorrect, and that the observations continued to increase; the authors attributed this in part to intense media attention, although such an effect is difficult to quantify. Forecasts were sensitive to the scaling factor used to relate model incidence to the data, much as our forecasts are influenced by *p*_*id*_, and in some cities, the cumulative incidence approached and even exceeded the total population. These difficulties – also present in our study – highlight the importance and complexity of selecting an appropriate observation model, due both the nature of the available data and the difficulty of relating infections in large, heterogeneous populations to a meta‐population model. Note that in both of these studies, a baseline ILI rate was subtracted from the weekly ILI estimates and negative values were set to zero, introducing a systematic positive bias in the weeks prior to each influenza season.

The same authors also employed EAKF and particle filter techniques to generate retrospective forecasts for Hong Kong, which has a humid subtropical climate and experiences irregular influenza epidemics.^30^ Observations were obtained by multiplying weekly ILI presentation rates at outpatient clinics by the proportion of collected specimens that tested positive for influenza. Both filtering techniques were able to predict peak timing up to 3 weeks in advance with comparable accuracy.

Dawson *et al*., building on an earlier study that used synthetic surveillance data,^6^ combined a particle filter with an SEIR infection model for the purposes of early outbreak detection, using a dynamic Bayesian network to assimilate disease surveillance observations provided by an agent‐based model.^31^ The emphasis on this study was the use of this method for early outbreak detection (e.g. in the case of a deliberate biological attack), rather than forecasting the progress of an extant epidemic. One of the drawbacks of dynamic Bayesian networks is the difficulty of defining the conditional probabilities for the network, but these can be informed by, for example sociological data for the relevant population. The use of this method as a forecasting tool was not fully explored, due to a different aim for the work, and the synthetic observation data were much more precise and complete than is possible for seasonal influenza outbreaks.

Interestingly, a simpler forecasting framework that used the ‘method of analogues’ to predict future influenza activity from past time‐series data was first published in 2003^32^ by Viboud *et al*. and has since seen ongoing use by the French GPs Sentinelles network (https://websenti.u707.jussieu.fr/sentiweb/). This method was shown to outperform autoregressive models, but the reliance upon past observed epidemic patterns to estimate future behaviour means that a comprehensive body of historical data is required.

More broadly, Lindström *et al*. presented a weighted ensemble modelling method that combines outputs from multiple models to allow for different degrees of model detail and different mechanistic assumptions about the infection process.^33^ This approach, adapted from climate modelling, was used to compare different interventions for the UK foot and mouth outbreak in 2001. The results were not evaluated as forecasts, but were instead used to explore the variation in projections obtained from a single model for different sets of model priors and control actions. Using this method with multiple models for forecasting purposes, one would expect to obtain forecasts that are less sensitive to individual model assumptions, although this could conceivably increase the forecast variance. We are not aware of any forecasting studies that have used multiple infection models, but combining meta‐population and individual‐based models for these purposes is an interesting possibility (contingent on sufficient demographic data).

### Meaning and implications

Predicting the time of the seasonal influenza epidemic peak – both accurately and precisely – several weeks in advance has clear benefits for public health decision making and resource allocation. In the urban Australian context, the results presented here suggest that such predictions can be obtained in the early stages of future influenza seasons, provided that an appropriate choice of observation model is made.

Good estimates of the net and peak burden – in terms of hospital and ICU admissions, in particular – are also critical for informing public health, and our results indicate that these quantities are significantly harder to predict due to model sensitivity (consistent with other studies^4^, ^5^, ^6^, ^31^). Accordingly, it is essential to determine how the accuracy of the epidemic size predictions obtained from these methods might be improved. This would, perhaps, be more readily addressed in an influenza *pandemic*, where ‘first few hundred’ studies would allow for rapid estimates of case severity and transmission characteristics.^34^

A critical requirement for obtaining reasonable forecasts is choosing an appropriate observation model (i.e. in form *and* parameter values). This is also, perhaps, the single greatest challenge in applying this methodology to epidemic forecasting and is particularly difficult in the face of surveillance systems whose characteristics vary over time (e.g. changes to the Google Flu Trends algorithm, recruitment of additional surveillance sites for syndromic systems, increased collection and testing of laboratory specimens for case notification systems). Such changes can occur from year to year, presenting a challenge for using data from previous influenza seasons to infer observation model parameters for future seasons, or can even occur during a single influenza season (e.g. as occurred during the 2009 pandemic) and require ‘on the fly’ tuning or estimation of observation model parameters.

### Further work

The most obvious and important question that remains unanswered is: how well did the retrospective forecasts correspond to the *true* seasonal influenza outbreaks in Melbourne for these calendar years? This is fundamentally very difficult to address, because the true infection process literally cannot be observed. However, as is true of all influenza forecasting studies to date, our forecasts were only compared to the same data source from which they were derived. Indeed, as recognised by Shaman and Karspeck, ‘we would prefer an observational estimate of weekly influenza infections that represents actual incidence as accurately as possible’.^4^

Critical to these efforts are robust methods for selecting observation models that can meaningfully relate observations to infection model dynamics. Where the observation systems are sufficiently complex or insufficiently characterised, it is pragmatic to select observation models based on their *phenomenological* description of the data (as in this study), rather than on being a mechanistic description of the poorly understood surveillance process.

Regardless, to identify optimal observation model parameter values (a key problem, as discussed above), a quantitative assessment of forecast quality is required. While such techniques have been developed in the atmospheric sciences,^35^ they typically require a statistically meaningful number of ‘true values’. In the context of influenza forecasting, the unit of forecasting is not a daily or weekly observation, but is instead an entire influenza outbreak; as Lindström *et al*. write: ‘epidemiological predictions suffer from lack of available data to assess model bias, and we propose that expert opinions will play a larger role than in other fields of research’. Thus, the only way to obtain sufficiently many samples of the ‘truth’ given the limitations of available data is to generate data *in silico* and add noise,^4^, ^6^ thereby approximating – at best – the true infection process in the target population. The development of methods applicable to the paucity of influenza epidemics for which data are available would therefore be of substantial importance. Investing in data collection efforts that greatly increase the population coverage (e.g. electronic health records) would permit substantial improvements to forecasting validation and performance.

Finally, additional data sources – comprising both syndromic surveillance and laboratory‐confirmed influenza notifications – are available for metropolitan Melbourne.^36^, ^37^, ^38^, ^39^ These systems are well‐characterised and have previously been analysed for spatiotemporal biases by our group.^40^ We are in the process of developing observation models for these and other systems, which will allow us to compare the forecasting quality obtained from each system. In comparison with Google Flu Trends, these systems provide more direct and transparent measures of disease activity in the community and will continue to operate indefinitely. Furthermore, by assimilating multiple data sources in the filtering process, the filter should be less susceptible to stochastic variation and noise in each system (thereby potentially avoiding *particle degeneracy*), with the result being more robust model forecasts.^41^

## Supporting information


**Table S1** Infection model priors and parameter values.
**Table S2** Particle filter parameters.
**Table S3** Observation model parameters.
**Figure S1** The ensemble forecast variances steadily decrease as the true epidemic peak is approached; the variance (log_10_) of the predicted size is shown on the left, the variance (log_10_) of the predicted time on the right.
**Figure S2** The accuracy of the peak timing forecasts (left, ±7, 10, 14 days) and the peak size forecasts (right, ±10%, 20%, 33%) increase as forecast variance decreases (i.e., as forecast precision increases).
**Figure S3** The accuracy of peak timing forecasts (left) and peak size forecasts (right) increase as the true epidemic peak is approached; dashed lines show 50% accuracy.
**Figure S4** A comparison of our original forecasts (*k* = 10) with forecasts generated using the Gaussian observation model of Shaman & Karspeck (“N S&K”, where variance is a function of the mean) and other Gaussian observation models with reduced variances (“N #1”… “N #4”).
**Figure S5** A comparison of our original forecasts (*k* = 100) with forecasts generated using the Gaussian observation model of Shaman & Karspeck (“N S&K”, where variance is a function of the mean) and other Gaussian observation models with reduced variances (“N #1” … “N #4”).
**Figure S6** A comparison of our original forecasts (*k* = 10) with forecasts generated using a Gaussian observation model (“N #4”) where the number of particles was increased five‐fold (“*N*_px_ = 18*K”*) and with a lower resampling threshold (“*N*_px_ = 18*K, N*_eff_ *>* 25%”).
**Figure S7** A comparison of our original forecasts (*k* = 100) with forecasts generated using a Gaussian observation model (“N #4”) where the number of particles was increased five‐fold (“*N*_*px*_ = 18*K”*) and with a lower resampling threshold (“*N*_px_ = 18*K, N*_eff_ *>* 25%”).
**Figure S8** A comparison of our original forecasts (*k* = 10) with forecasts generated using Gaussian observation models with constant variances (“*N* (σ = 100)” and “*N* (σ = 170)”), and with a lower resampling threshold (“*N* (σ = 170)*, N*_eff_ *>* 25%”).
**Figure S9** A comparison of our original forecasts (*k* = 100) with forecasts generated using Gaussian observation models with constant variances (“*N* (σ = 100)” and “*N* (σ = 170)”), and with a lower resampling threshold (“*N*(σ = 170), *N*_eff_ *>* 25%”).
**Figure S10** A comparison of our original forecasts (*k* = 10) with forecasts generated using the original model where the number of particles was increased five‐fold (“*N*_*px*_ = 18*K*”) and where the resampling threshold was decreased (“*N*_eff_ *>* 25%” and “*N*_eff_ *>* 50%”).
**Figure S11** A comparison of our original forecasts (*k* = 100) with forecasts generated using the original model where the number of particles was increased five‐fold (“*N*_*px*_ = 18*K*”) and where the resampling threshold was decreased (“*N*_eff_ *>* 25%” and “*N*_eff_ *>* 50%”).
**Figure S12** A comparison of our original forecasts (*k* = 10) with forecasts generated using the Gaussian observation model of Shaman & Karspeck (“N S&K”, where variance is a function of the mean) and other Gaussian observation models with reduced variances (“N #1” … “N #4”).
**Figure S13** A comparison of our original forecasts (*k* = 100) with forecasts generated using the Gaussian observation model of Shaman & Karspeck (“N S&K”, where variance is a function of the mean) and other Gaussian observation models with reduced variances (“N #1” … “N #4”).
**Figure S14** A comparison of our original forecasts (*k* = 10) with forecasts generated using a Gaussian observation model (“N #4”) where the number of particles was increased five‐fold (“*N*_*px*_ = 18*K”*) and with a lower resampling threshold (“*N*_*px*_ = 18*K, N*_eff_ *>* 25%”).
**Figure S15** A comparison of our original forecasts (*k* = 100) with forecasts generated using a Gaussian observation model (“N #4”) where the number of particles was increased five‐fold (“*N*_*px*_ = 18*K*”) and with a lower resampling threshold (“*N*_*px*_ = 18*K, N*_eff_ *>* 25%”).
**Figure S16** A comparison of our original forecasts (*k* = 10) with forecasts generated using Gaussian observation models with constant variances (“*N* (σ = 100)” and “*N* (σ = 170)”), and with a lower resampling threshold (“*N* (σ = 170), *N*_eff_ *>* 25%”).
**Figure S17** A comparison of our original forecasts (*k* = 100) with forecasts generated using Gaussian observation models with constant variances (“*N* (σ = 100)” and “*N* (σ = 170)”), and with a lower resampling threshold (“*N* (σ = 170), *N*_eff_ *>* 25%”).
**Figure S18** A comparison of our original forecasts (*k* = 10) with forecasts generated using the original model where the number of particles was increased five‐fold (“*N*_*px*_ = 18*K”*) and where the resampling threshold was decreased (“*N*_eff_ *>* 25%” and “*N*_eff_ *>* 50%”).
**Figure S19** A comparison of our original forecasts (*k* = 100) with forecasts generated using the original model where the number of particles was increased five‐fold (“*N*_*px*_ = 18*K*”) and where the resampling threshold was decreased (“*N*_eff_ *>* 25%” and “*N*_eff_ *>* 50%”).

## References

[1] Chan T‐C , King C‐C , Yen M‐Y , Chiang P‐H , Huang C‐S , Hsiao CK . Probabilistic daily ILI syndromic surveillance with a spatio‐temporal Bayesian hierarchical model. PLoS ONE 2010; 5:e11626. 20661275 10.1371/journal.pone.0011626 PMC2905374

[2] Nsoesie EO , Brownstein JS , Ramakrishnan N , Marathe MV . A systematic review of studies on forecasting the dynamics of influenza outbreaks. Influenza Other Respir Viruses 2014; 8:309–316. 24373466 10.1111/irv.12226 PMC4181479

[3] Ong JBS , Chen MI‐C , Cook AR *et al* Real‐time epidemic monitoring and forecasting of H1N1‐2009 using influenza‐like illness from general practice and family doctor clinics in Singapore. PLoS ONE 2010; 5:e10036. 20418945 10.1371/journal.pone.0010036 PMC2854682

[4] Shaman J , Karspeck A . Forecasting seasonal outbreaks of influenza. Proc Natl Acad Sci USA 2012; 109:20425–20430. 23184969 10.1073/pnas.1208772109 PMC3528592

[5] Shaman J , Karspeck A , Yang W , Tamerius J , Lipsitch M . Real‐time influenza forecasts during the 2012‐2013 season. Nat Commun 2013; 4:2837. 24302074 10.1038/ncomms3837 PMC3873365

[6] Skvortsov A , Ristic B . Monitoring and prediction of an epidemic outbreak using syndromic observations. Math Biosci 2012; 240:12–19. 22705339 10.1016/j.mbs.2012.05.010

[7] Axelsen JB , Yaari R , Grenfell BT , Stone L . Multiannual forecasting of seasonal influenza dynamics reveals climatic and evolutionary drivers. Proc Natl Acad Sci 2014; 111:9538–9542. 24979763 10.1073/pnas.1321656111 PMC4084473

[8] Neri FM , Cook AR , Gibson GJ , Gottwald TR , Gilligan CA . Bayesian analysis for inference of an emerging epidemic: citrus canker in urban landscapes. PLoS Comput Biol 2014; 10:e1003587. 24762851 10.1371/journal.pcbi.1003587 PMC3998883

[9] Ginsberg J , Mohebbi MH , Patel RS , Brammer L , Smolinski MS , Brilliant L . Detecting influenza epidemics using search engine query data. Nature 2008; 457:1012–1014. 19020500 10.1038/nature07634

[10] Flu Trends Team . The next chapter for flu trends. 2015 Available at http://googleresearch.blogspot.com.au/2015/08/the-next-chapter-for-flu-trends.html (Accessed 2015‐08‐21).

[11] Yang W , Karspeck A , Shaman J . Comparison of filtering methods for the modeling and retrospective forecasting of influenza epidemics. PLoS Comput Biol 2014; 10:e1003583. 24762780 10.1371/journal.pcbi.1003583 PMC3998879

[12] McCaw JM , McVernon J , McBryde ES , Mathews JD . Influenza: accounting for prior immunity. Science 2009; 325:1071. 19713508 10.1126/science.325_1071a

[13] Katriel G , Stone L . Pandemic dynamics and the breakdown of herd immunity. Marshall JAR, editor. PLoS ONE 2010; 5:e9565. 20300617 10.1371/journal.pone.0009565 PMC2837721

[14] Kitagawa G . Monte Carlo filter and smoother for non‐Gaussian nonlinear state space models. J Comput Graph Stat 1996; 5:1–25.

[15] Department of Health & Human Services . 2013 local government area profiles. 2013.

[16] Lindén A , Mäntyniemi S . Using the negative binomial distribution to model overdispersion in ecological count data. Ecology 2011; 92:1414–1421. 21870615 10.1890/10-1831.1

[17] Ver Hoef JM , Boveng PL . Quasi‐Poisson vs. negative binomial regression: how should we model overdispersed count data? Ecology 2007; 88:2766–2772. 18051645 10.1890/07-0043.1

[18] White GC , Bennetts RE . Analysis of frequency count data using the negative binomial distribution. Ecology 1996; 77:2549.

[19] Alexander N , Moyeed R , Stander J . Spatial modelling of individual‐level parasite counts using the negative binomial distribution. Biostatistics. 2000; 1:453–463. 12933567 10.1093/biostatistics/1.4.453

[20] Lloyd‐Smith JO , Schreiber SJ , Kopp PE , Getz WM . Superspreading and the effect of individual variation on disease emergence. Nature 2005; 438:355–359. 16292310 10.1038/nature04153 PMC7094981

[21] Mathews JD , McCaw CT , McVernon J , McBryde ES , McCaw JM . A biological model for influenza transmission: pandemic planning implications of asymptomatic infection and immunity. Monk N, editor. PLoS ONE 2007; 2:e1220. 18043733 10.1371/journal.pone.0001220 PMC2080757

[22] Cauchemez S , Ferguson NM . Likelihood‐based estimation of continuous‐time epidemic models from time‐series data: application to measles transmission in London. J R Soc Interface 2008; 5:885–897. 18174112 10.1098/rsif.2007.1292 PMC2607466

[23] Bolker BM . Ecological models and data in R. Princeton, NJ: Princeton University Press; 2008.

[24] Olson DR , Konty KJ , Paladini M , Viboud C , Simonsen L . Reassessing Google Flu Trends data for detection of seasonal and pandemic influenza: a comparative epidemiological study at three geographic scales. PLoS Comput Biol 2013; 9:e1003256. 24146603 10.1371/journal.pcbi.1003256 PMC3798275

[25] Lazer D , Kennedy R , King G , Vespignani A . The parable of Google Flu: traps in big data analysis. Science 2014; 343:1203–1205. 24626916 10.1126/science.1248506

[26] Domnich A , Panatto D , Signori A , Lai PL , Gasparini R , Amicizia D . Age‐related differences in the accuracy of web query‐based predictions of influenza‐like illness. PLoS ONE 2015; 10:e0127754. 26011418 10.1371/journal.pone.0127754 PMC4444192

[27] Owen R , Barr IG , Pengilley A *et al* Annual report of the National Influenza Surveillance Scheme. 2007. Commun Dis Intell Q Rep 2008; 32:208–226. 18767420 10.33321/cdi.2008.32.19

[28] Carlson SJ , Dalton CB , Butler MT , Fejsa J , Elvidge E , Durrheim DN . Flutracking weekly online community survey of influenza‐like illness annual report 2011 and 2012. Commun Dis Intell Q Rep 2013; 37:E398–E406. 24882237 10.33321/cdi.2013.37.51

[29] NNDSS Annual Report Writing Group . Australia's notifiable disease status, 2011: Annual report of the National Notifiable Diseases Surveillance System. Commun Dis Intell Q Rep 2013; 37:E313–E393. 24882235 10.33321/cdi.2013.37.49

[30] Yang W , Cowling BJ , Lau EHY , Shaman J . Forecasting influenza epidemics in Hong Kong. PLoS Comput Biol 2015; 11:e1004383. 26226185 10.1371/journal.pcbi.1004383 PMC4520691

[31] Dawson P , Gailis R , Meehan A . Detecting disease outbreaks using a combined Bayesian network and particle filter approach. J Theor Biol 2015; 370:171–183. 25637764 10.1016/j.jtbi.2015.01.023

[32] Viboud C , Boëlle PY , Carrat F , Valleron A , Flahault A . Prediction of the spread of influenza epidemics by the method of analogues. Am J Epidemiol 2003; 158:996–1006. 14607808 10.1093/aje/kwg239

[33] Lindström T , Tildesley M , Webb C . A Bayesian ensemble approach for epidemiological projections. Kosakovsky Pond SL, editor. PLoS Comput Biol 2015; 11:e1004187. 25927892 10.1371/journal.pcbi.1004187 PMC4415763

[34] McLean E , Pebody RG , Campbell C *et al* Pandemic (H1N1) 2009 influenza in the UK: clinical and epidemiological findings from the first few hundred (FF100) cases. Epidemiol Infect 2010; 138:1531–1541. 20594381 10.1017/S0950268810001366

[35] Wilks DS . Statistical methods in the atmospheric sciences. Amsterdam:Elsevier, 2006.

[36] Kelly H , Murphy A , Leong W *et al* Laboratory‐supported influenza surveillance in Victorian sentinel general practices. Commun Dis Intell 2000; 24:379–383. 11225381 10.33321/cdi.2000.24.68

[37] Turner J , Kelly H . A medical locum service as a site for sentinel influenza surveillance. Euro Surveill 2005; 10:96–98. 15879645

[38] Parrella A , Dalton CB , Pearce R , Litt JCB , Stocks N . ASPREN surveillance system for influenza‐like illness ‐ a comparison with FluTracking and the National Notifiable Diseases Surveillance System. Aust Fam Physician 2009; 38:932–936. 19893847

[39] Kaczmarek M , Owen R , Barr IG . Annual report of the National Influenza Surveillance Scheme, 2008. Commun Dis Intell Q Rep 2010; 34:8–22. 20521494 10.33321/cdi.2010.34.2

[40] Thomas EG , McCaw JM , Kelly HA , Grant KA , McVernon J . Quantifying differences in the epidemic curves from three influenza surveillance systems: a nonlinear regression analysis. Epidemiol Infect 2014; 143:1–13. 24759447 10.1017/S0950268814000764 PMC9206772

[41] De Angelis D , Presanis AM , Birrell PJ , Tomba GS , House T . Four key challenges in infectious disease modelling using data from multiple sources. Epidemics. 2015; 10:83–87. 25843390 10.1016/j.epidem.2014.09.004 PMC4383805

