# Supplementary material for: Forecasting influenza outbreak dynamics in Melbourne from Internet search query surveillance data
Source: Influenza Other Respir Viruses. 2016 Mar 7;10(4):314–23. doi: 10.1111/irv.12376 (PMC4910172; doi:10.1111/irv.12376)
Supplement: Supplementary file 1 — Table S1 Infection model priors and parameter values. Table S2 Particle filter parameters. Table S3 Observation model parameters. Figure S1 The ensemble forecast variances steadily decrease as the true epidemic peak is approached; the variance (log10) of the predicted size is shown on the left, the variance (log10) of the predicted time on the right. Figure S2 The accuracy of the peak timing forecasts (left, ±7, 10, 14 days) and the peak size forecasts (right, ±10%, 20%, 33%) increase as forecast variance decreases (i.e., as forecast precision increases). Figure S3 The accuracy of peak timing forecasts (left) and peak size forecasts (right) increase as the true epidemic peak is approached; dashed lines show 50% accuracy. Figure S4 A comparison of our original forecasts (k = 10) with forecasts generated using the Gaussian observation model of Shaman & Karspeck (“N S&K”, where variance is a function of the mean) and other Gaussian observation models with reduced variances (“N #1”… “N #4”). Figure S5 A comparison of our original forecasts (k = 100) with forecasts generated using the Gaussian observation model of Shaman & Karspeck (“N S&K”, where variance is a function of the mean) and other Gaussian observation models with reduced variances (“N #1” … “N #4”). Figure S6 A comparison of our original forecasts (k = 10) with forecasts generated using a Gaussian observation model (“N #4”) where the number of particles was increased five‐fold (“N px = 18K”) and with a lower resampling threshold (“N px = 18K, N eff > 25%”). Figure S7 A comparison of our original forecasts (k = 100) with forecasts generated using a Gaussian observation model (“N #4”) where the number of particles was increased five‐fold (“N px = 18K”) and with a lower resampling threshold (“N px = 18K, N eff > 25%”). Figure S8 A comparison of our original forecasts (k = 10) with forecasts generated using Gaussian observation models with constant variances (“N (σ = 100)” and “N (σ = 170)”), and with a lower res [file IRV-10-314-s001.pdf]

Supplementary Material

S1 Disease model

Each particle encompasses the state variables and model parameters for an SEIR compartment model (Eq. S1) and are initially sampled from the prior distributions listed in Table S1. The flows between compartments are defined by Eqs. S2–S5; stochastic noise is included in these flows (Eqs. S6–S9) and in the model parameters (Eq. S10) as per Skvortsov and Ristic (2012). The entire population is assumed to be susceptible at $t = 0$ and an initial exposure occurs with daily probability p_{seed} (Eq. S11).

$$\mathbf{x_t} = [S(t), E(t), I(t), R(t), \alpha(t), \beta(t), \gamma(t), \eta(t)]^T \tag{S1}$$

$$\frac{dS}{dt} = -\alpha(S^\eta)I - \zeta_S - \theta_{\text{seed}} \tag{S2}$$

$$\frac{dE}{dt} = \alpha(S^\eta)I + \zeta_S + \theta_{\text{seed}} - \beta E - \zeta_E \tag{S3}$$

$$\frac{dI}{dt} = \beta E + \zeta_E - \gamma I - \zeta_I \tag{S4}$$

$$\frac{dR}{dt} = \gamma I + \zeta_I \tag{S5}$$

$$\zeta_{(S,E,I)} \sim \mathcal{N}(\mu = 0, \sigma = \sigma_{(S,E,I)}) \tag{S6}$$

$$\sigma_S = \kappa_F \cdot \sqrt{\alpha S^\eta I} \cdot N^{-1/2} \tag{S7}$$

$$\sigma_E = \kappa_F \cdot \sqrt{\beta E} \cdot N^{-1/2} \tag{S8}$$

$$\sigma_I = \kappa_F \cdot \sqrt{\gamma I} \cdot N^{-1/2} \tag{S9}$$

$$\frac{d\alpha}{dt}, \frac{d\beta}{dt}, \frac{d\gamma}{dt}, \frac{d\eta}{dt} \sim \mathcal{N}(\mu = 0, \sigma = \kappa_P) \tag{S10}$$

$$\theta_{\text{seed}} = \begin{cases} 1 & \text{if } S(t) = 1 \text{ and } \theta(t) < p_{\text{seed}} \\ 0 & \text{otherwise} \end{cases} \tag{S11}$$

	Meaning	Value
$S(0)$	Initial susceptible population	N
$E(0)$	Initial exposed population	0
$I(0)$	Initial infectious population	0
$R(0)$	Initial recovered population	0
$\alpha(0)$	Force of infection	$\sim \mathcal{U}(0.2, 1)$
$\beta(0)$	Incubation period (days^{-1})	$\sim [\mathcal{U}(0.5, 3)]^{-1}$
$\gamma(0)$	Infectious period (days^{-1})	$\sim [\mathcal{U}(1, 3)]^{-1}$
$\eta(0)$	Inhomogeneous social mixing exponent	$\sim \mathcal{U}(1, 2)$
p_{seed}	Daily probability of seeding an initial exposure	1/36
$\theta(t)$	Stochastic variable for seeding an initial exposure	$\sim \mathcal{U}(0, 1)$
κ_F	Scaling factor for the compartment-flow noise	0.025
κ_P	Scaling factor for the parameter noise	0.005

Table S1: Infection model priors and parameter values.

S2 Particle filter

Particles are initially assigned uniform weights w_i (Eq. S12), which are subsequently adjusted in response to each observation $\mathbf{y_t}$ (Eq. S13) and normalised so as to sum to one (Eq. S14). When the *effective* number of particles (Eq. S15) drops below the threshold N_{min} (Table S2) the particles are *resampled* in proportion to their weights; this is done using the systematic (deterministic) method, as described by Kitagawa (1996, see appendix).

$$w_i(0) = (N_{\text{px}})^{-1} \tag{S12}$$

$$w'_i(t \mid \mathbf{y_t}) = w_i(t-1) \cdot P(\mathbf{y_t} \mid \mathbf{x_t^i}; k) \tag{S13}$$

$$w_i(t \mid \mathbf{y_t}) = w'_i(t) \cdot \left(\sum_{j=1}^{N_{\text{px}}} w'_j(t) \right)^{-1} \tag{S14}$$

$$N_{\text{eff}}(t) = \left(\sum_{j=1}^{N_{\text{px}}} \left[w'_j(t) \right]^2 \right)^{-1} \tag{S15}$$

	Meaning	Value
N_{px}	Number of particles	3,600
N_{min}	Minimum threshold for *effective* particles	$0.75 \times N_{\text{px}}$

Table S2: Particle filter parameters.

S3 Observation model

Google Flu Trends reports ILI prevalence as *integer counts* of ILI cases per 100,000 GP visits, and so we can express the daily probability of an individual presenting with ILI in the absence of influenza (p_{bg}) as a function of the (imposed) background ILI rate B_R (Table S3) and the daily number of GP visits per individual (v_{daily}) (Eq. S16). The value for v_{daily} was obtained from the reported annual rate of 5,615 GP attendances per 1,000 population (Department of Health & Human Services, 2013).

The probability that an infected individual will visit a GP and be identified as having ILI (p_{id}) is a parameter of the observation model (Table S3). The probability that an individual will be identified as an ILI case over some time interval $[t-\Delta, t]$ is the sum of two independent events (Eq. S17): becoming infectious (p_{inf}) and being identified (p_{id}), or not becoming infectious but presenting with an ILI. The probability of becoming infectious is defined as the fraction of the population that became infectious (i.e., transitioned from state E to state I) during the time interval (Eq. S18), and subsumes both symptomatic and asymptomatic infections. In the absence of reliable data to the contrary, both types of infection are assumed to be identically infectious. The observation probability (p_{id}) therefore represents the probability of an infection being symptomatic *and* observed.

A negative binomial distribution is used to define the likelihood of obtaining an ILI presentation rate $\mathbf{y_t}$ from a given particle $\mathbf{x_t}$ (Eq. S19). We use the parameter κ to convert ILI incidence from cases per 100,000 GP visits to population incidence (Eq. S20). The *dispersion parameter* k (Eq. S21) controls the variance: as k increases the variance decreases and the distribution approaches the Poisson.

$$p_{\text{bg}} = v_{\text{daily}} \cdot B_R \times 10^{-5} \tag{S16}$$

$$p_{\text{ili}}(t, \Delta) = p_{\text{inf}}(t, \Delta) \cdot p_{\text{id}} + [1 - p_{\text{inf}}(t, \Delta)] \cdot \Delta \cdot p_{\text{bg}} \tag{S17}$$

$$p_{\text{inf}}(t, \Delta) = \frac{S(t-\Delta) + E(t-\Delta) - S(t) - E(t)}{N} \tag{S18}$$

$$P(\mathbf{y_t} \mid \mathbf{x_t}; k) = \frac{\Gamma([\kappa \cdot \mathbf{y_t}] + k)}{\Gamma(k) \cdot [\kappa \cdot \mathbf{y_t}]!} \cdot (p_{\text{k}})^k \cdot (1 - p_{\text{k}})^{[\kappa \cdot \mathbf{y_t}]} \tag{S19}$$

$$\kappa = \frac{N \cdot v_{\text{daily}} \cdot 7}{100{,}000} \tag{S20}$$

$$p_{\text{k}} = \frac{k}{k + N \cdot p_{\text{ili}}} \tag{S21}$$

	Meaning	Value
N	Melbourne metropolitan population	4,108,541
v_{daily}	Daily number of GP visits per individual	0.0154
Δ	Observation period (days)	7
B_R	Background ILI rate per 100,000 visits	*varies*; 200–350
k	Dispersion parameter	*varies;* 10^0–10^3
p_{id}	Probability of ILI observation per infection	*varies*; 0.0025–0.1000

Table S3: Observation model parameters.

S4 Forecast variance and accuracy

Here we show how the forecast variance and accuracy change over the weeks leading up to the observed epidemic peak for each calendar year.

As shown in Figure S1, the variance of both the peak size and timing forecasts decrease substantially as the forecasting date approaches the date of the true epidemic peak. Note that the y-axis is logarithmic and differs in scale between the two plots, since the peak time forecasts are more precise than the peak size forecasts.

Forecast accuracy increases as the forecast variance decreases (Figure S2) and so the accuracy increases as the true peak is approached (Figure S3). For example, when using a threshold of ± 10 days as the definition of an "accurate" prediction of the peak timing (equivalent to ± 1 week, when aggregating dates into weekly bins) it can be seen that in 4 of the calendar years under consideration, more than 50% of the weighted forecasts accurately predicted the timing of the peak 5 weeks in advance.

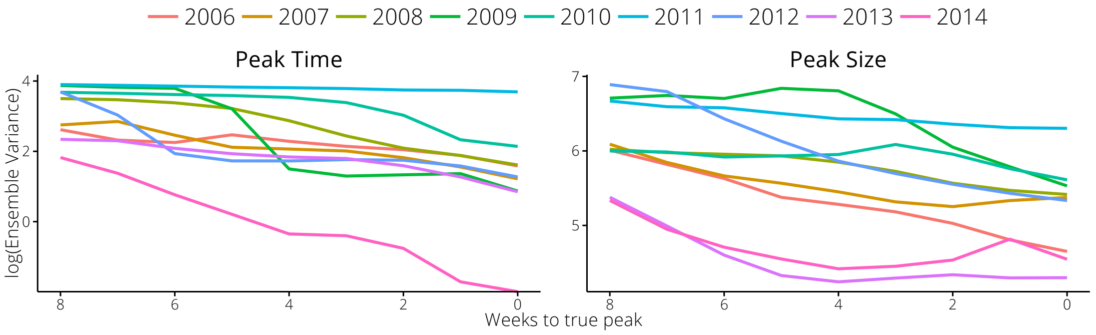

Figure S1: The ensemble forecast variances steadily decrease as the true epidemic peak is approached; the variance ($\log_{10}$) of the predicted size is shown on the left, the variance ($\log_{10}$) of the predicted time on the right. Results are shown for $B_R = 300$, $p_{\mathrm{id}} = 0.05$ and $k = 10$; three years are excluded on the grounds that the epidemic peak was either very small (2010), absent (2011), or occurred after an earlier, smaller peak (2014).

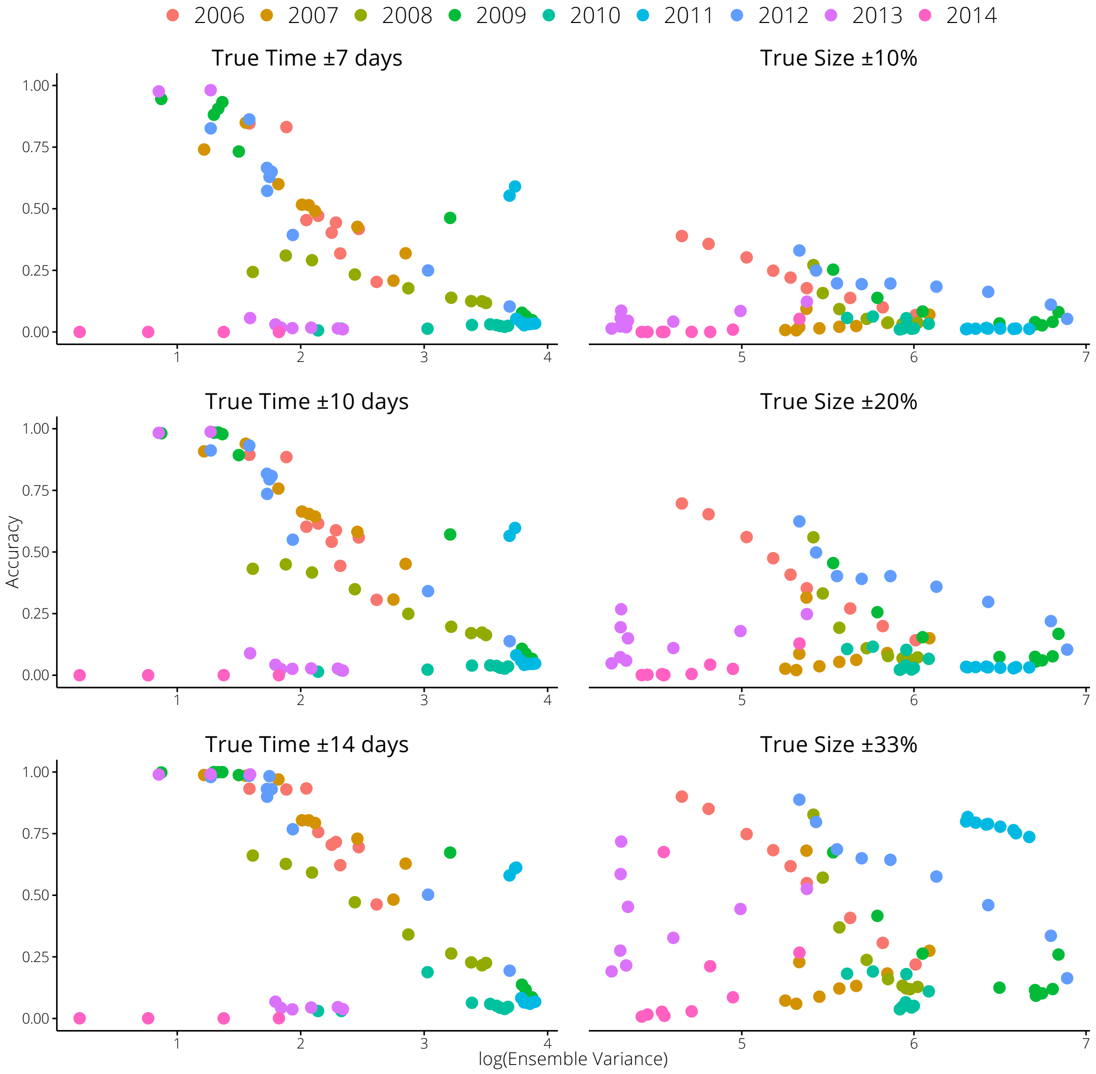

Figure S2: The accuracy of the peak timing forecasts (left, $\pm 7, 10, 14$ days) and the peak size forecasts (right, $\pm 10\%, 20\%, 33\%$) increase as forecast variance decreases (i.e., as forecast precision increases). Forecast accuracy is shown for three tolerance levels, relative to the true peak time (left) and peak size (right). Results are shown for the same forecasts as in Figure S1.

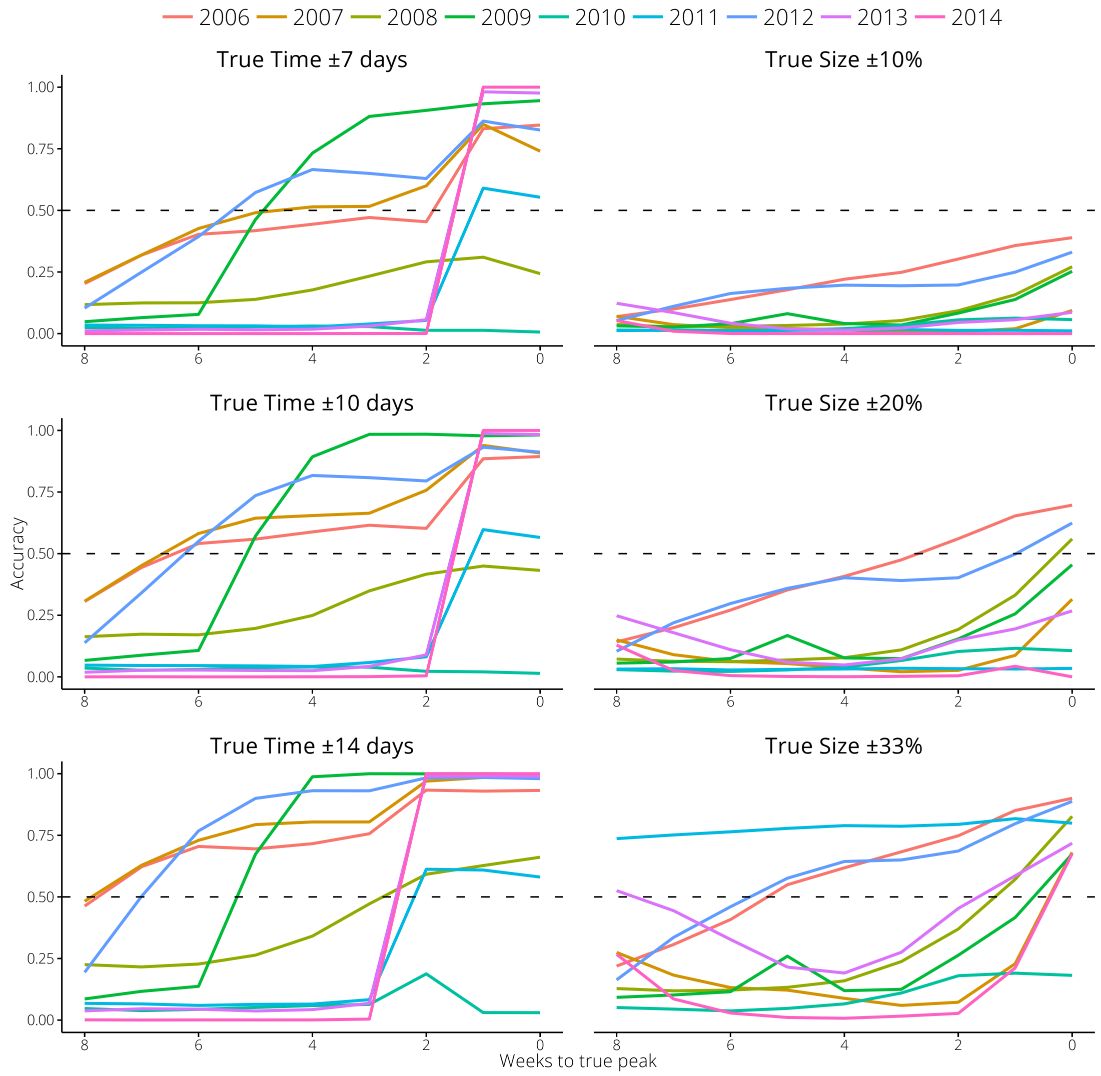

Figure S3: The accuracy of peak timing forecasts (left) and peak size forecasts (right) increase as the true epidemic peak is approached; dashed lines show 50% accuracy. Results are shown for the same forecasts as in Figure S1.

S5 Particle filter robustness

In an effort to avoid particle degeneracy (as observed, e.g., in the 2014 forecasts for $k = 10$ and $k = 100$), we explored a number of approaches to improve filter robustness.

We tested the normal distribution observation model used by Shaman and Karspeck (2012), where the variance was a function of the mean ("N S&K", Figures S4–S5). However the variance was sufficiently large to reduce forecast performance in every year except 2013 and 2014. We also explored using similar observation models where the magnitude of the variance was reduced ("N #1" ... "N #4", Figures S4–S5) and observed that this did not improve forecasting performance in 2013 and 2014.

Increasing the number of particles five-fold ("$N_{\text{px}} = 18K$") and/or decreasing the resampling threshold ("$N_{\text{eff}} > 25\%$") did not improve forecasting performance in 2013 and 2014 (as shown for the "N #4" observation model in Figures S6–S7).

We also tested two normal distributions where the variance was estimated using data from the early phase of the season: first, using all values < 450 ($\sigma = 100$); and second, using all values < 800 ($\sigma = 170$), but neither improved forecasting performance in 2013 and 2014, even when the resampling threshold was decreased ("$N_{\text{eff}} > 25\%$"), shown in Figures S8–S9).

We also tested the performance of our original (negative binomial) observation model when the number of particles was increased five-fold ("$N_{\text{px}} = 18K$") and when the resampling threshold was reduced ("$N_{\text{eff}} > 25\%$" and "$N_{\text{eff}} > 50\%$") but, as shown in Figures S10–S11, this did not improve forecasting performance in 2013 and 2014.

For each of these modifications to the particle filter, we also plotted the resulting accuracy of the peak timing forecasts over the weeks prior to the true peak (with a tolerance of ±10 days) against the accuracy of the original method ("Original (k=10)" and "Original (k=100)") in Figures S12–S19.

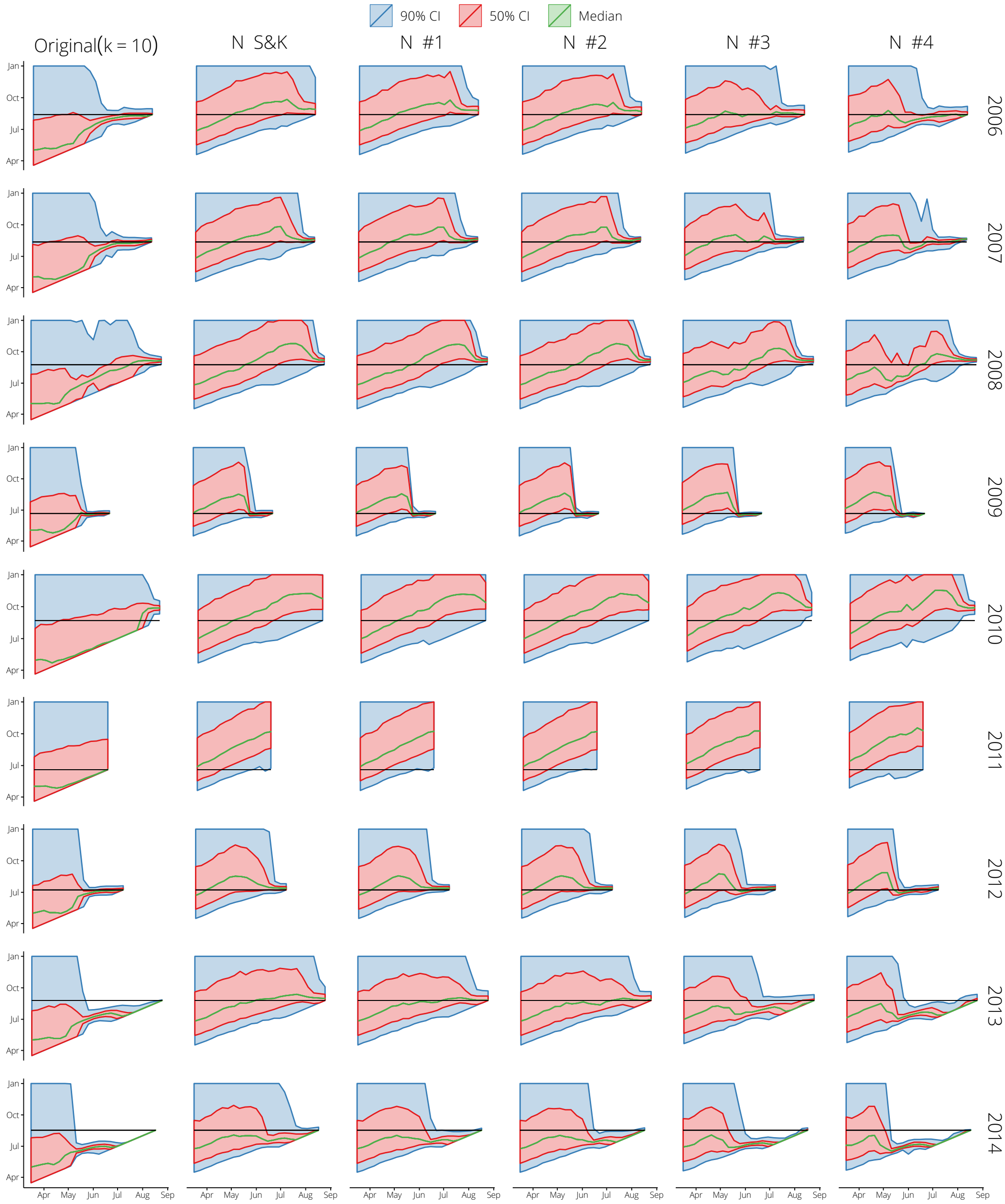

Figure S4: A comparison of our original forecasts ($k = 10$) with forecasts generated using the Gaussian observation model of Shaman & Karspeck ("N S&K", where variance is a function of the mean) and other Gaussian observation models with reduced variances ("N #1" … "N #4").

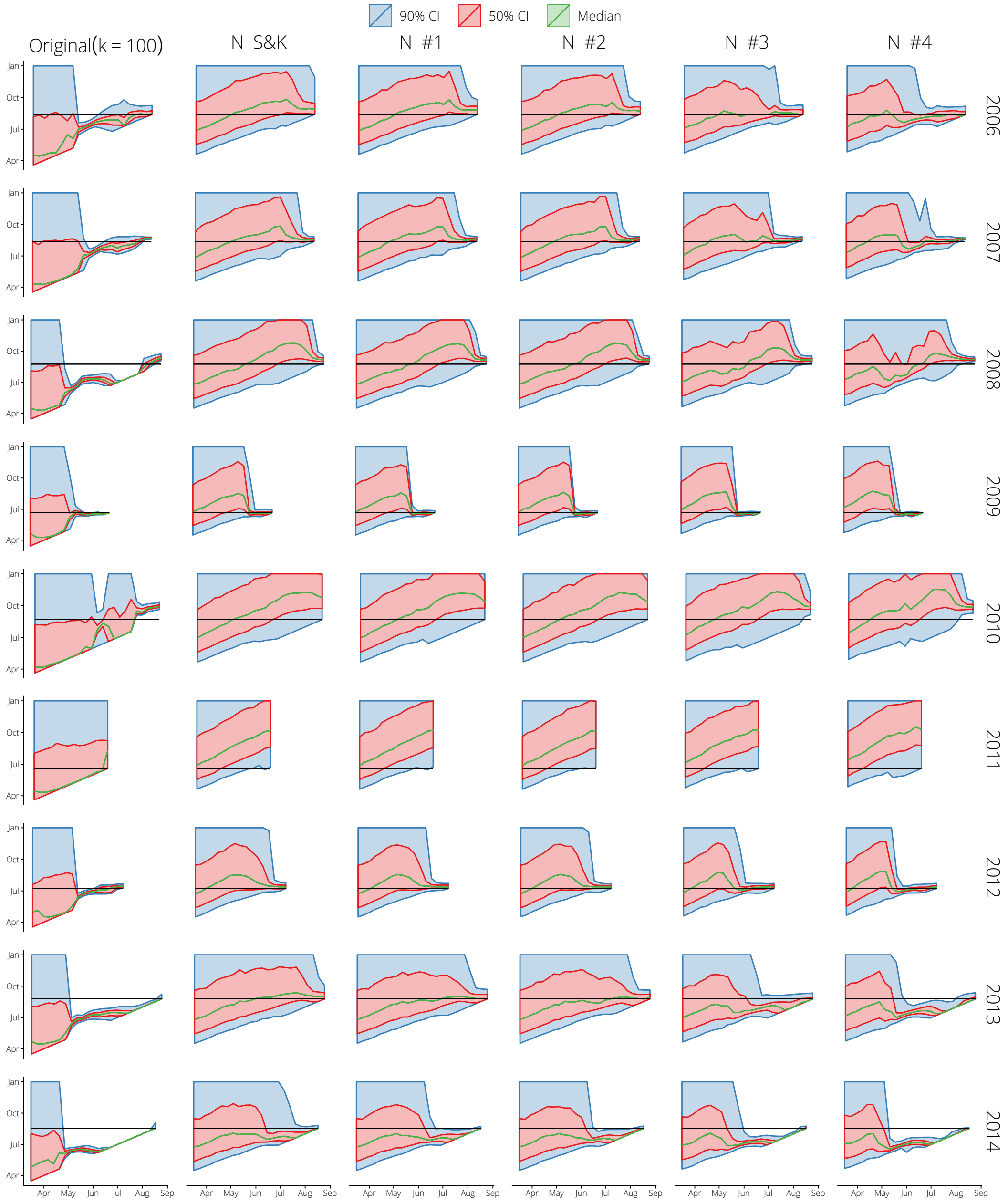

Figure S5: A comparison of our original forecasts ($k = 100$) with forecasts generated using the Gaussian observation model of Shaman & Karspeck ("N S&K", where variance is a function of the mean) and other Gaussian observation models with reduced variances ("N #1" … "N #4").

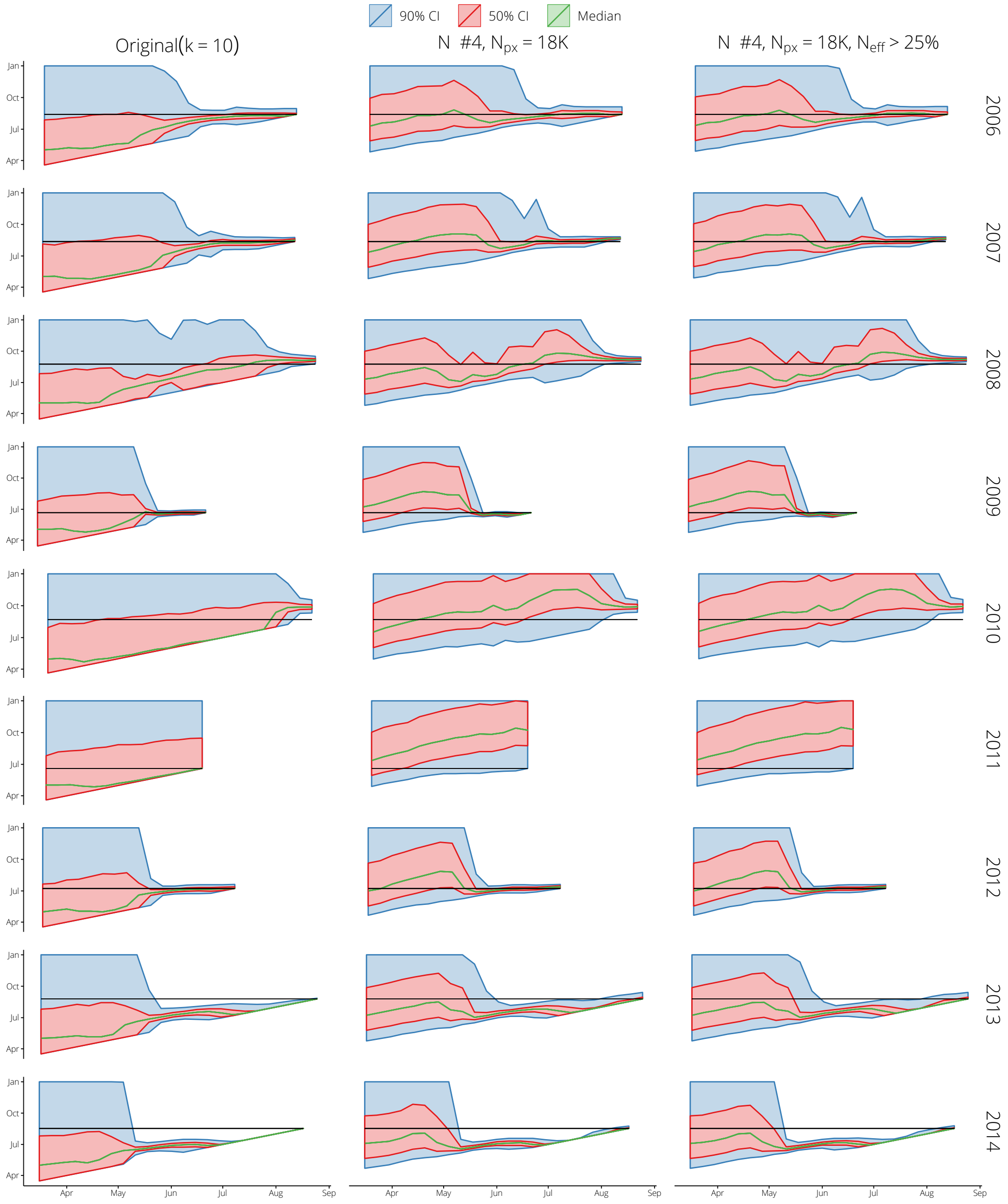

Figure S6: A comparison of our original forecasts ($k = 10$) with forecasts generated using a Gaussian observation model ("N #4") where the number of particles was increased five-fold ("$N_{px} = 18K$") and with a lower resampling threshold ("$N_{px} = 18K, N_{\text{eff}} > 25\%$").

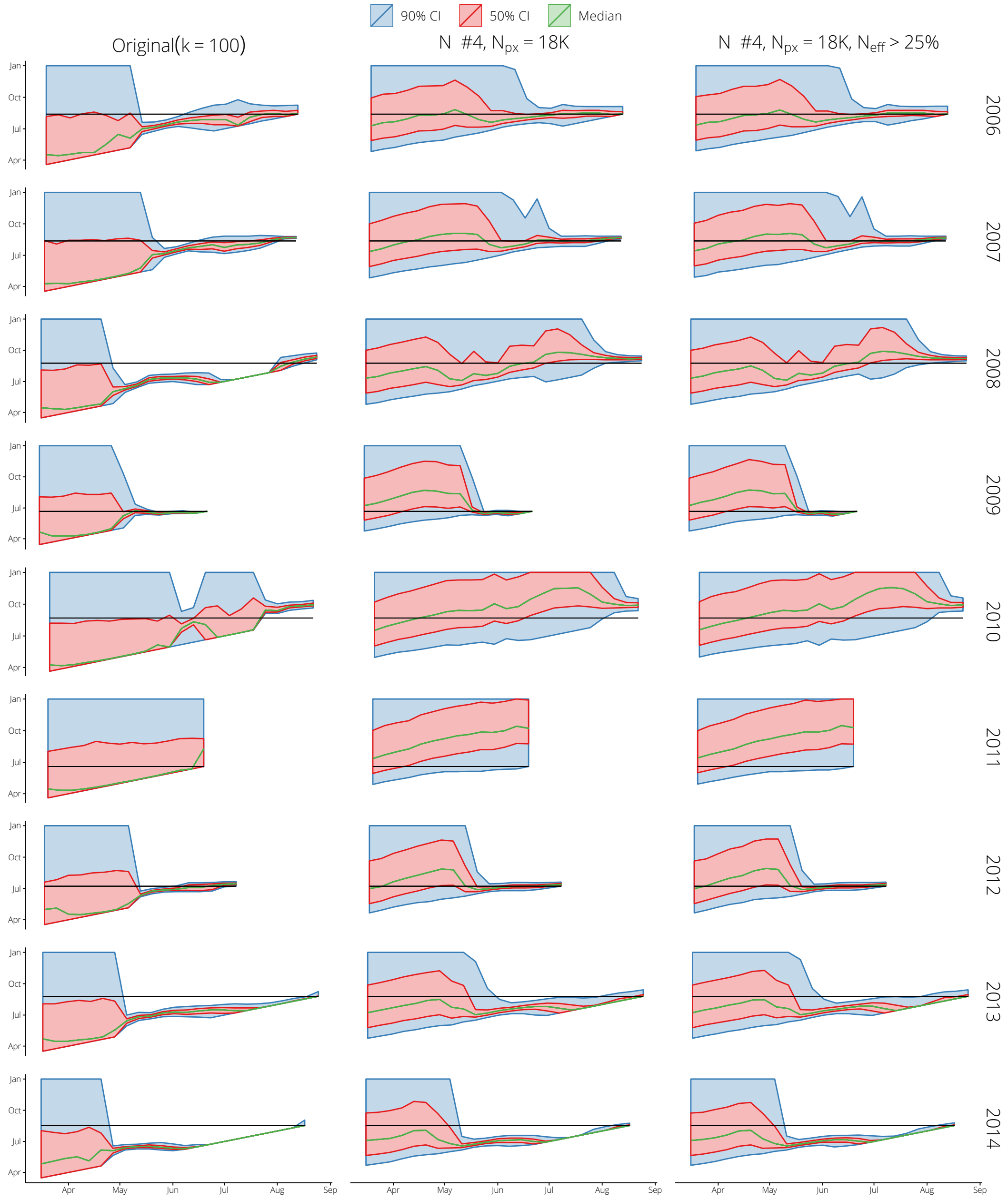

Figure S7: A comparison of our original forecasts ($k = 100$) with forecasts generated using a Gaussian observation model ("N #4") where the number of particles was increased five-fold ("$N_{px} = 18K$") and with a lower resampling threshold ("$N_{px} = 18K, N_{\text{eff}} > 25\%$").

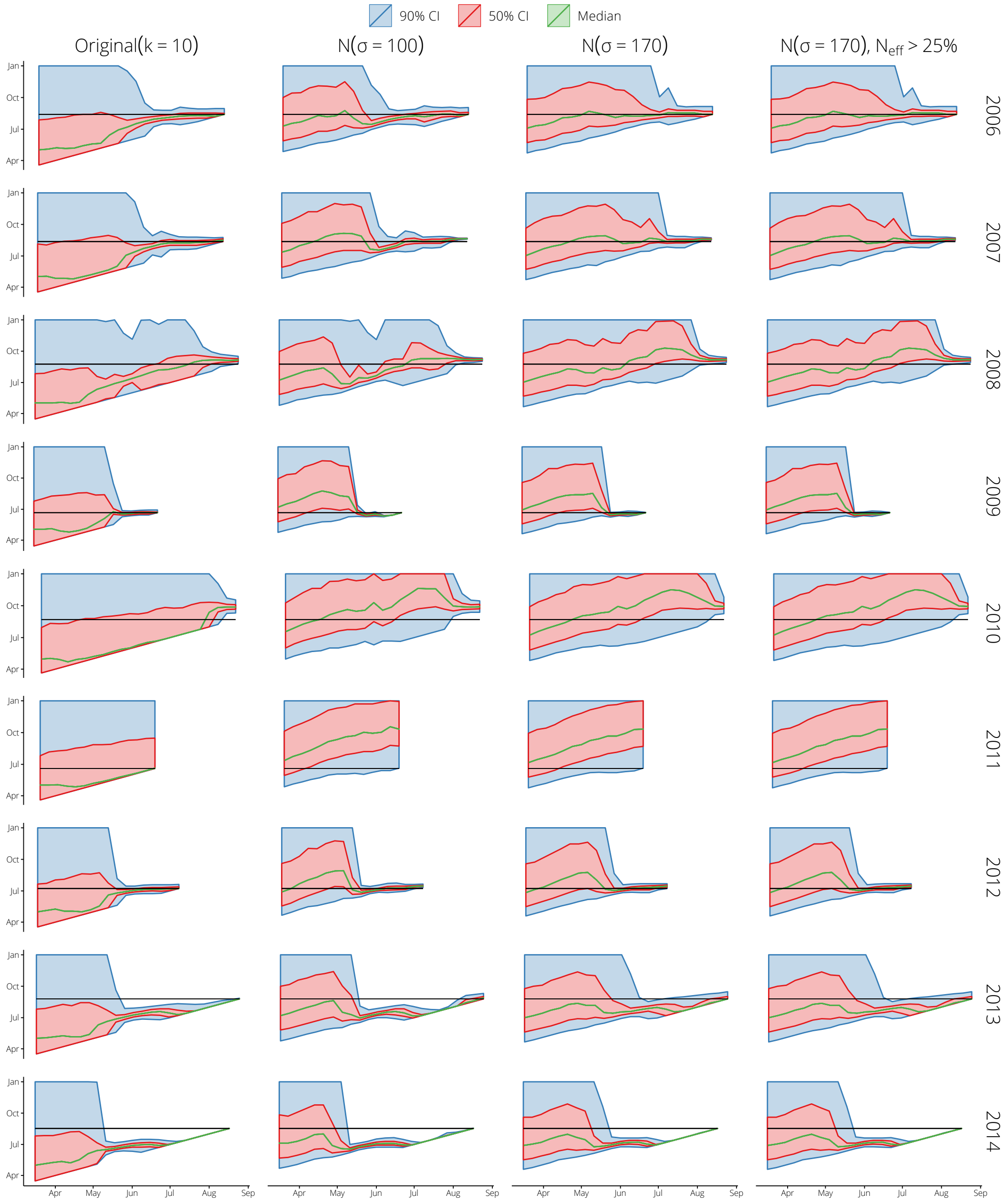

Figure S8: A comparison of our original forecasts ($k = 10$) with forecasts generated using Gaussian observation models with constant variances ("$N(\sigma = 100)$" and "$N(\sigma = 170)$"), and with a lower resampling threshold ("$N(\sigma = 170), N_{\text{eff}} > 25\%$").

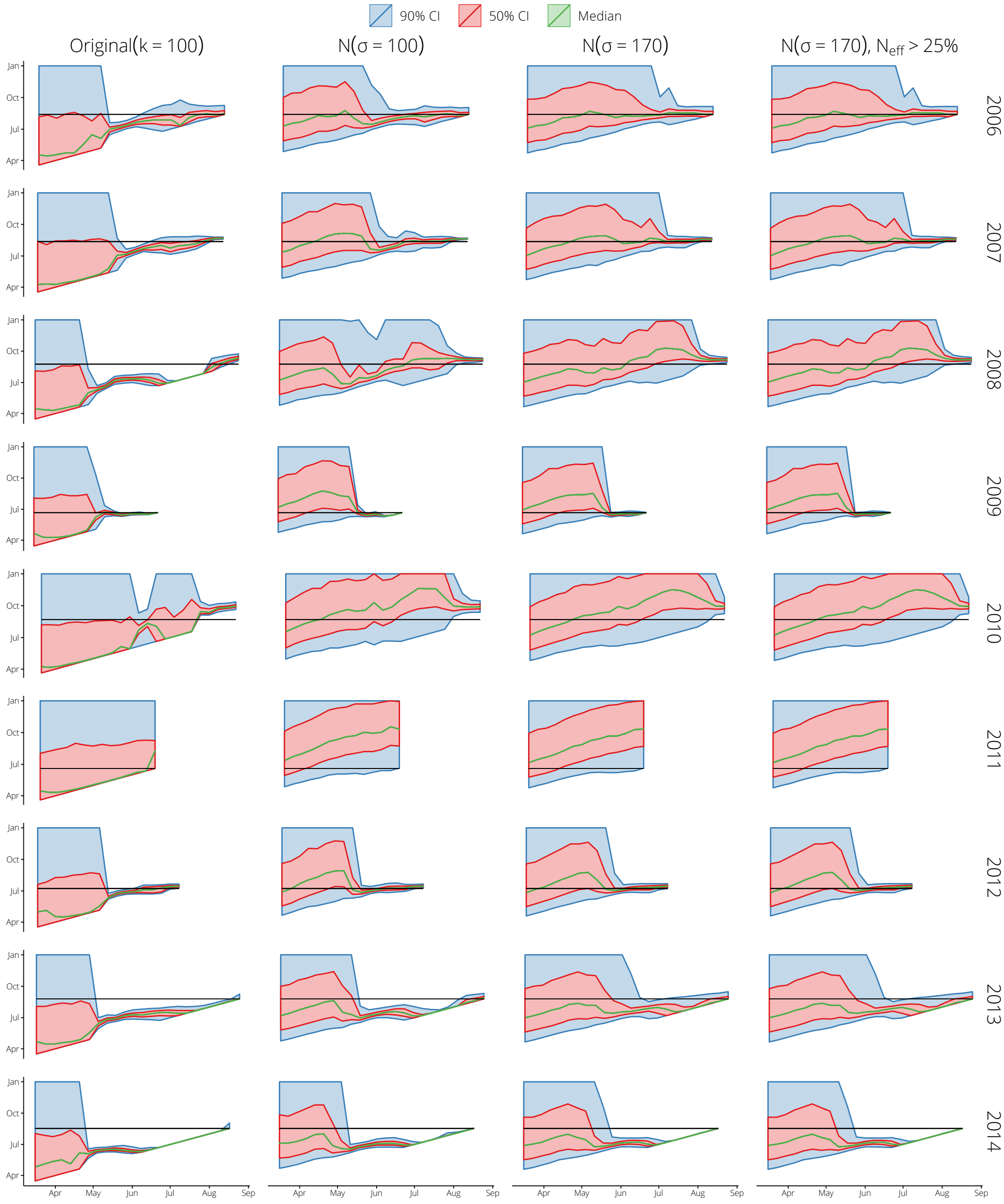

Figure S9: A comparison of our original forecasts ($k = 100$) with forecasts generated using Gaussian observation models with constant variances ("$N(\sigma = 100)$" and "$N(\sigma = 170)$"), and with a lower resampling threshold ("$N(\sigma = 170), N_{\text{eff}} > 25\%$").

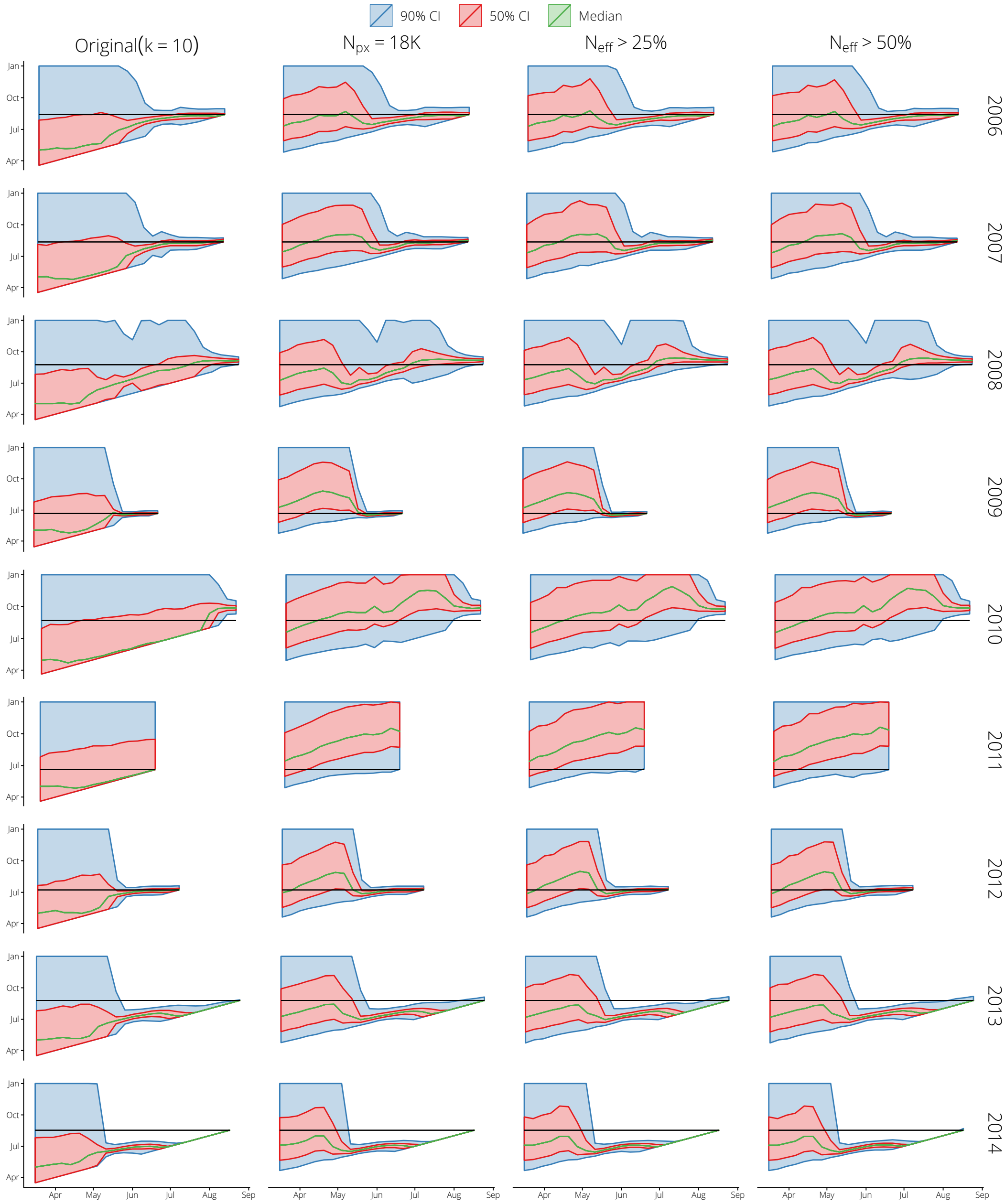

Figure S10: A comparison of our original forecasts ($k = 10$) with forecasts generated using the original model where the number of particles was increased five-fold ("$N_{px} = 18K$") and where the resampling threshold was decreased ("$N_{\text{eff}} > 25\%$" and "$N_{\text{eff}} > 50\%$").

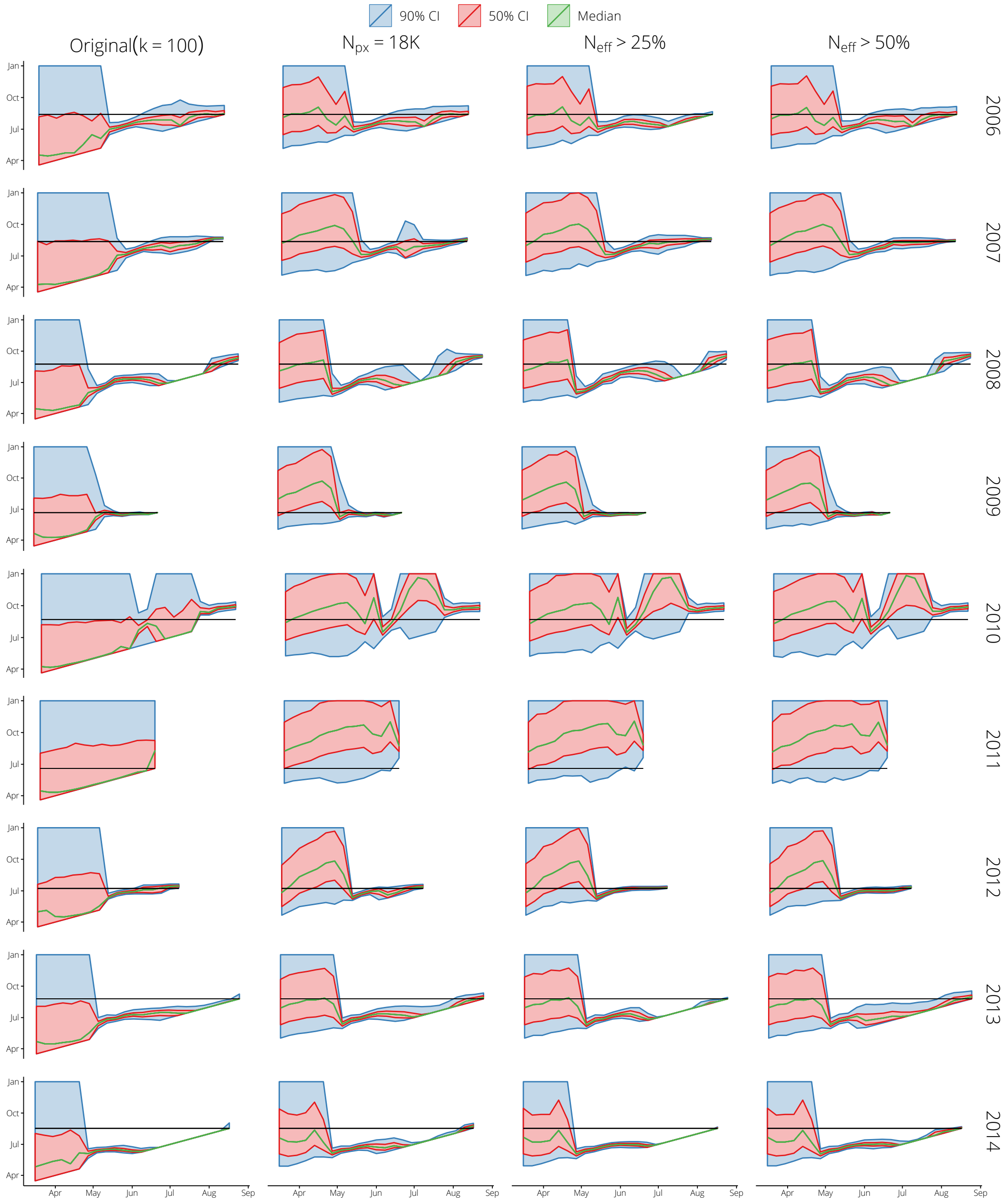

Figure S11: A comparison of our original forecasts ($k = 100$) with forecasts generated using the original model where the number of particles was increased five-fold ("$N_{px} = 18K$") and where the resampling threshold was decreased ("$N_{\text{eff}} > 25\%$" and "$N_{\text{eff}} > 50\%$").

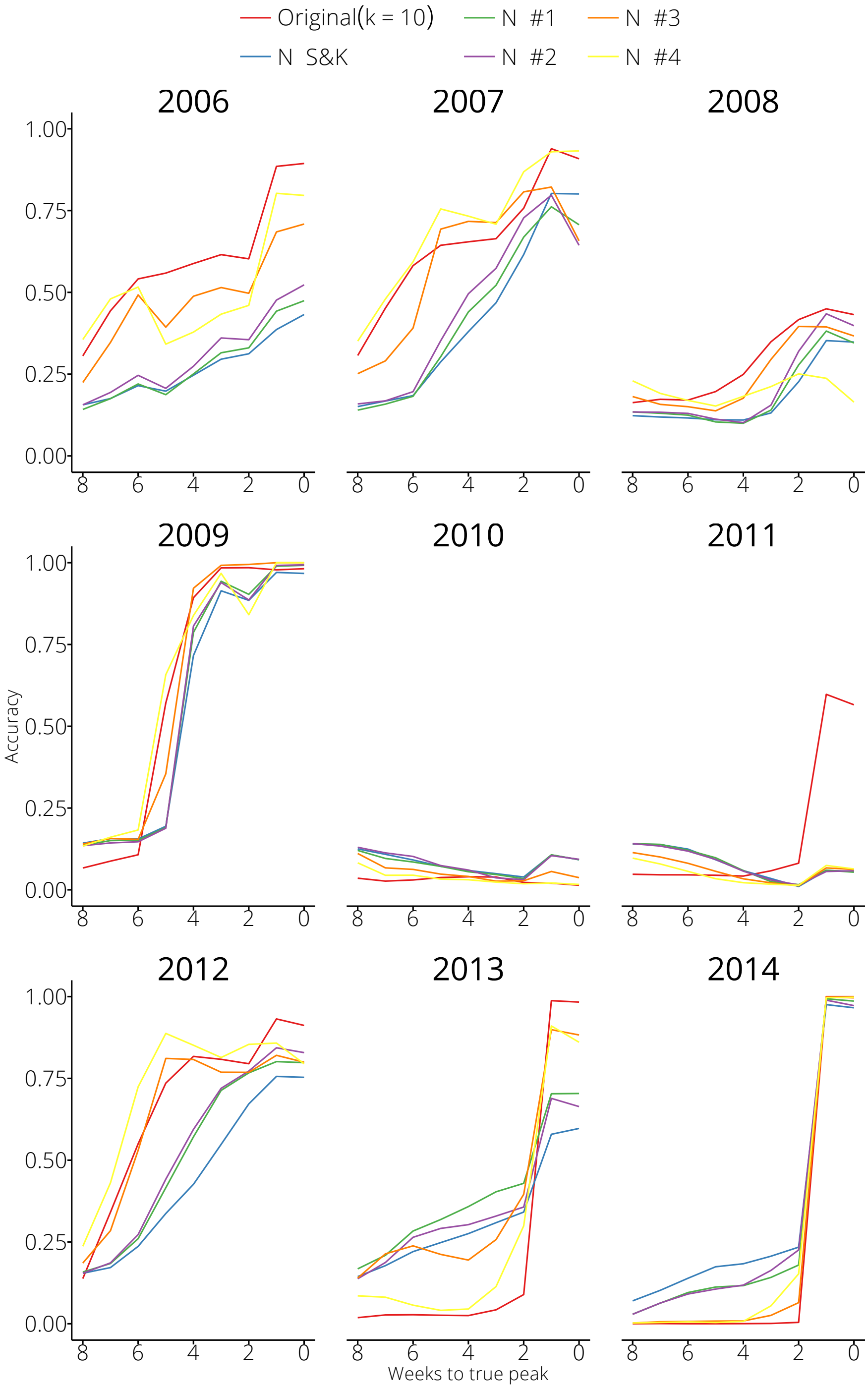

Figure S12: A comparison of our original forecasts ($k = 10$) with forecasts generated using the Gaussian observation model of Shaman & Karspeck ("N S&K", where variance is a function of the mean) and other Gaussian observation models with reduced variances ("N #1" … "N #4").

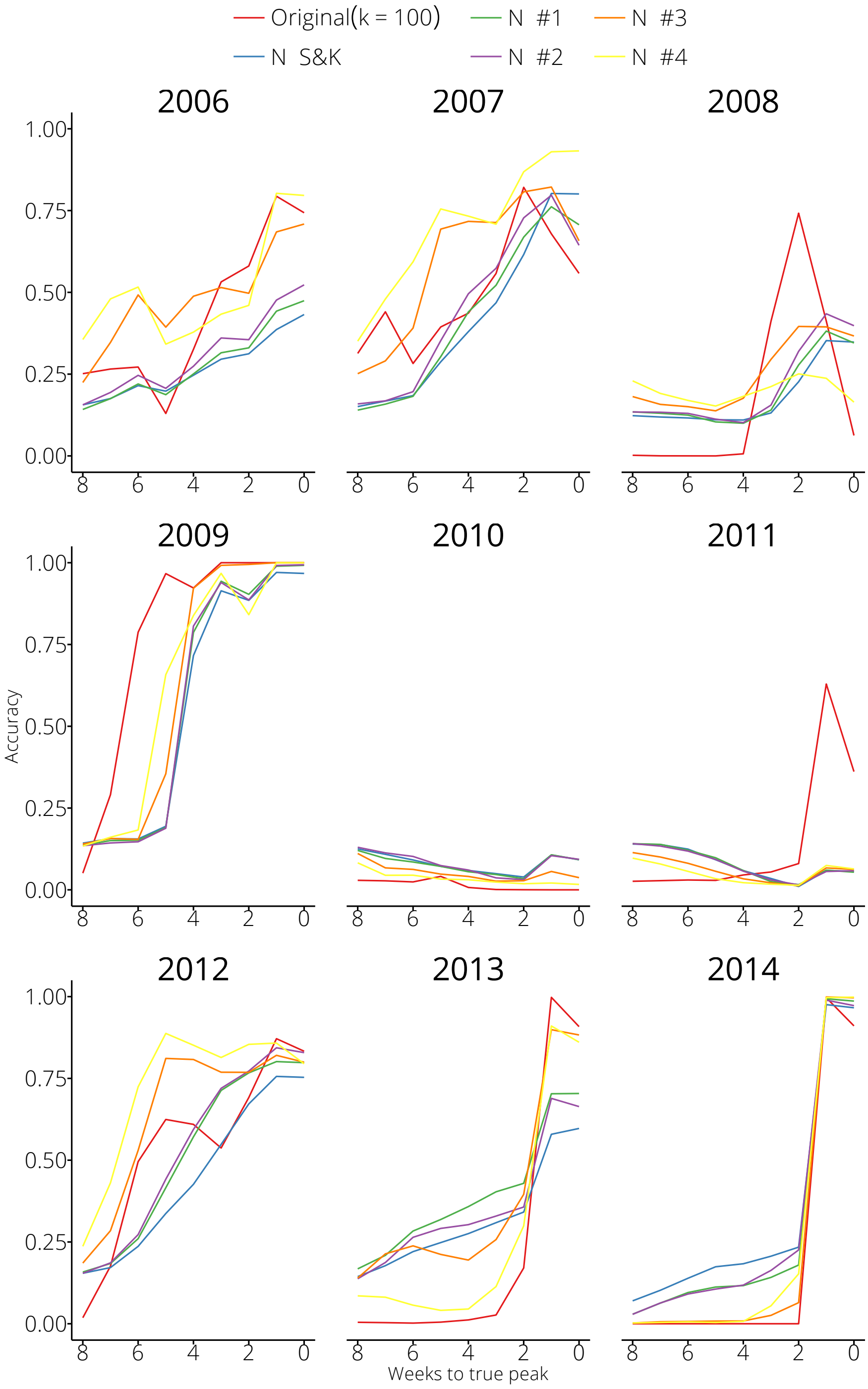

Figure S13: A comparison of our original forecasts ($k = 100$) with forecasts generated using the Gaussian observation model of Shaman & Karspeck ("N S&K", where variance is a function of the mean) and other Gaussian observation models with reduced variances ("N #1" ... "N #4").

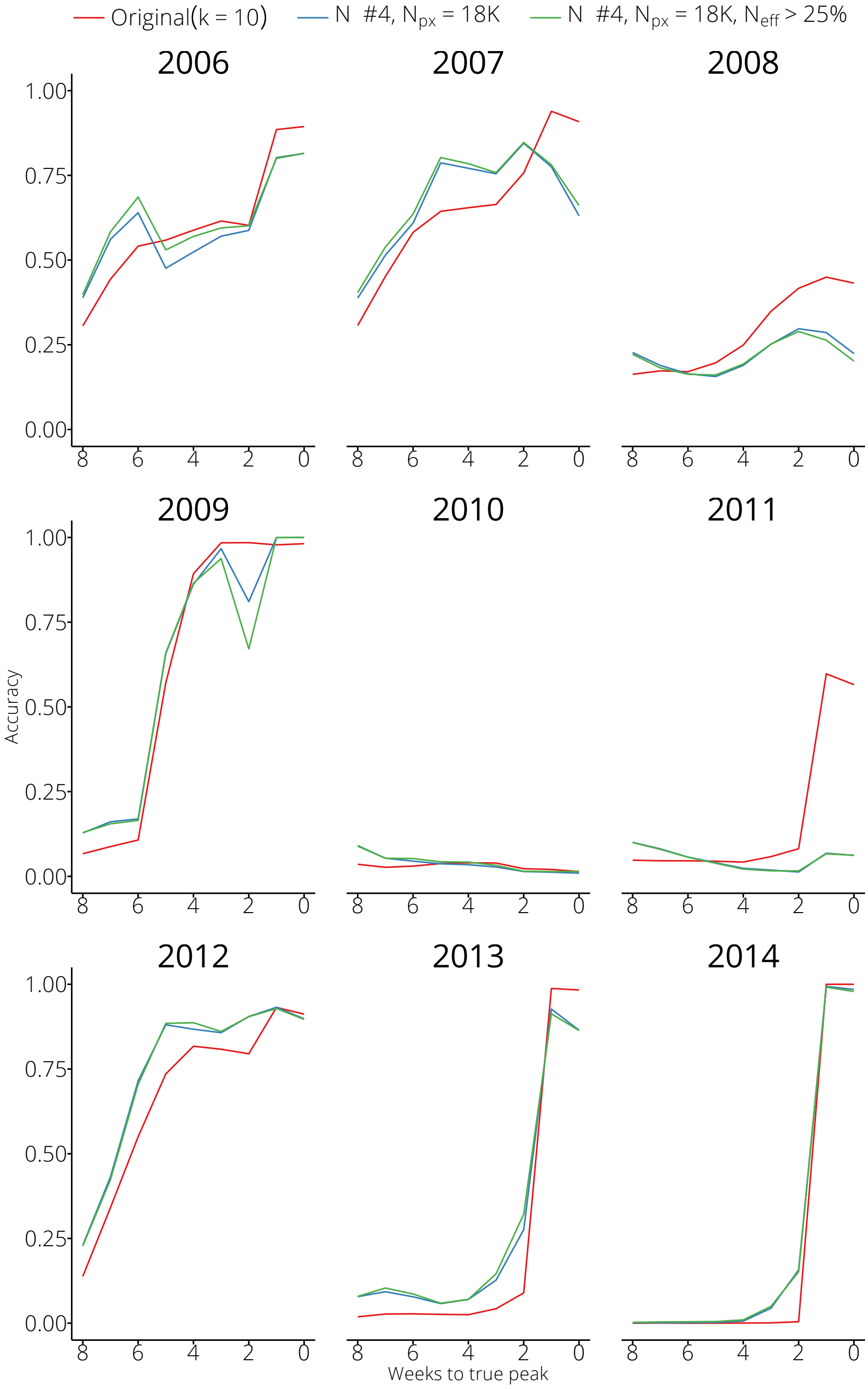

Figure S14: A comparison of our original forecasts ($k = 10$) with forecasts generated using a Gaussian observation model ("N #4") where the number of particles was increased five-fold ("$N_{px} = 18K$") and with a lower resampling threshold ("$N_{px} = 18K, N_{\text{eff}} > 25\%$").

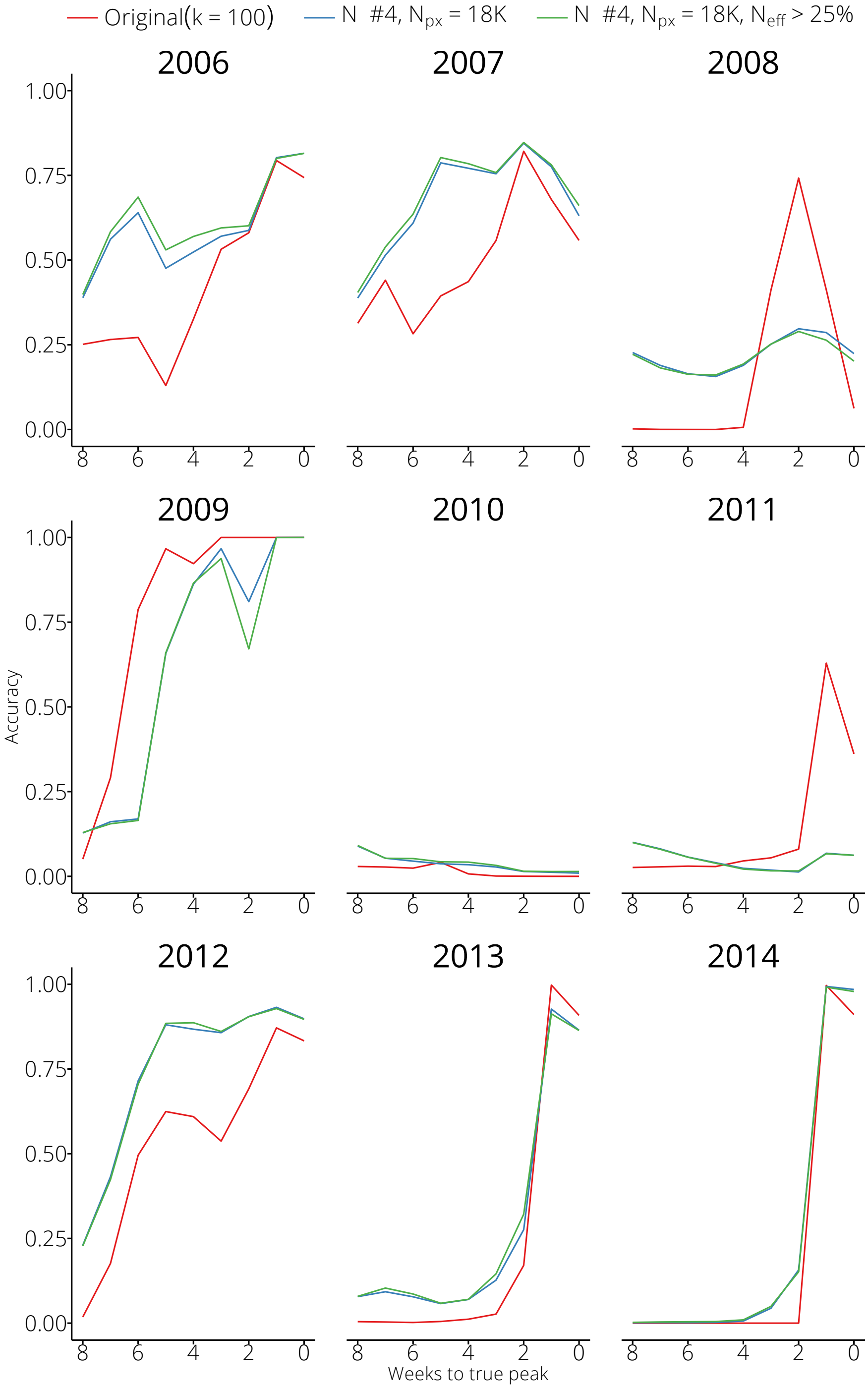

Figure S15: A comparison of our original forecasts ($k = 100$) with forecasts generated using a Gaussian observation model ("N #4") where the number of particles was increased five-fold ("$N_{px} = 18K$") and with a lower resampling threshold ("$N_{px} = 18K, N_{\text{eff}} > 25\%$").

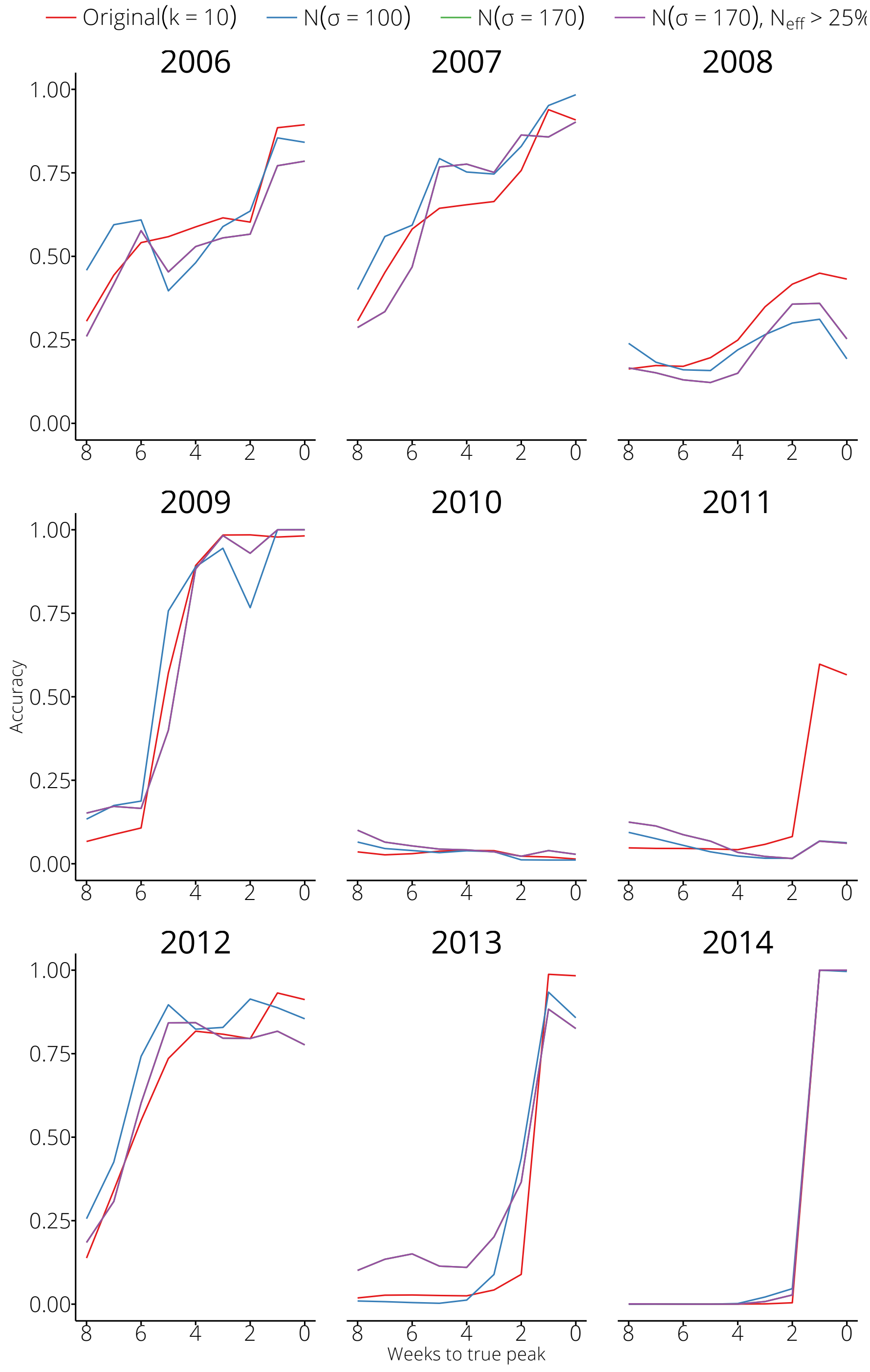

Figure S16: A comparison of our original forecasts ($k = 10$) with forecasts generated using Gaussian observation models with constant variances ("$N(\sigma = 100)$" and "$N(\sigma = 170)$"), and with a lower resampling threshold ("$N(\sigma = 170)$, $N_{\text{eff}} > 25\%$").

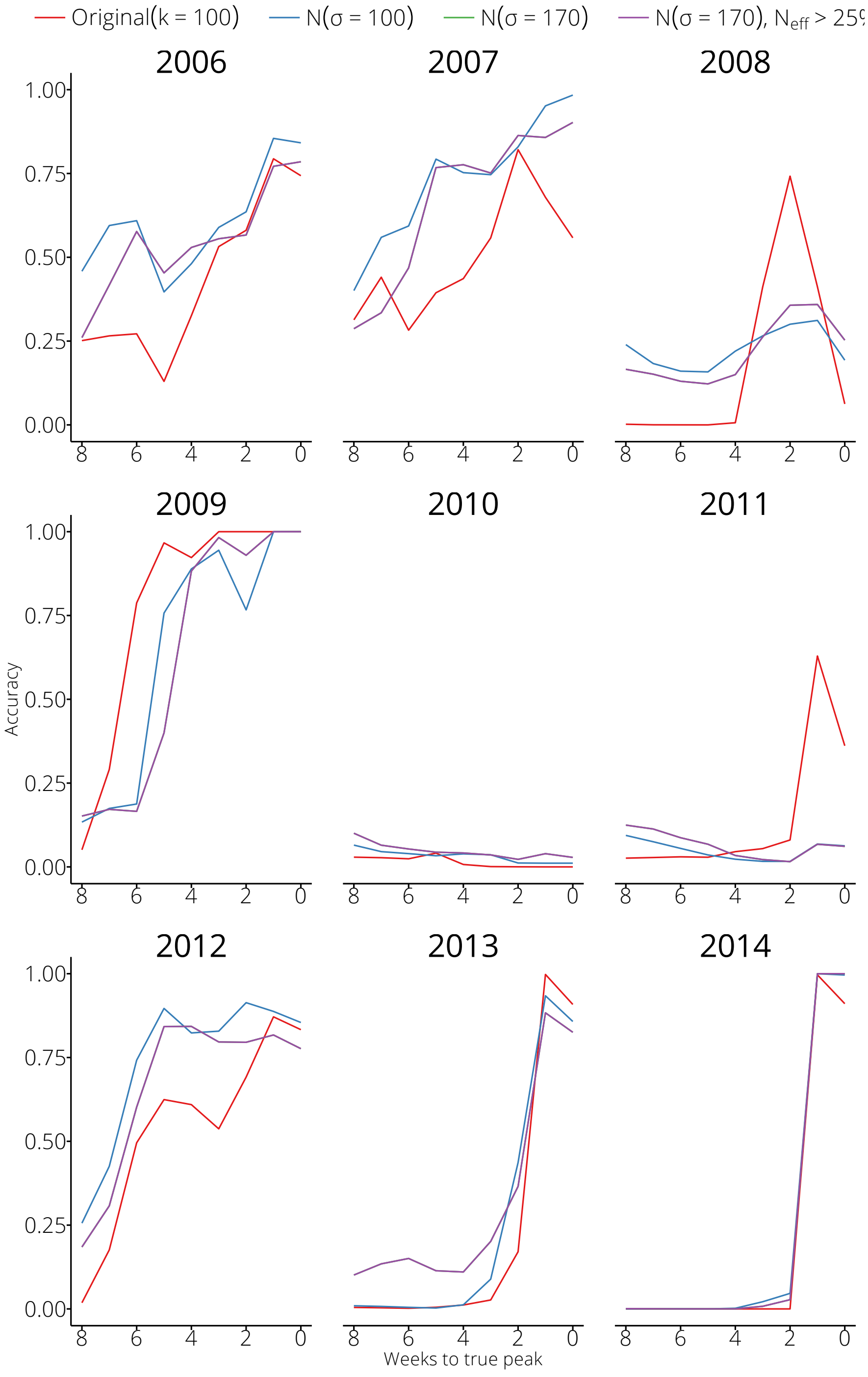

Figure S17: A comparison of our original forecasts ($k = 100$) with forecasts generated using Gaussian observation models with constant variances ("$N(\sigma = 100)$" and "$N(\sigma = 170)$"), and with a lower resampling threshold ("$N(\sigma = 170)$, $N_{\text{eff}} >$ 25%").

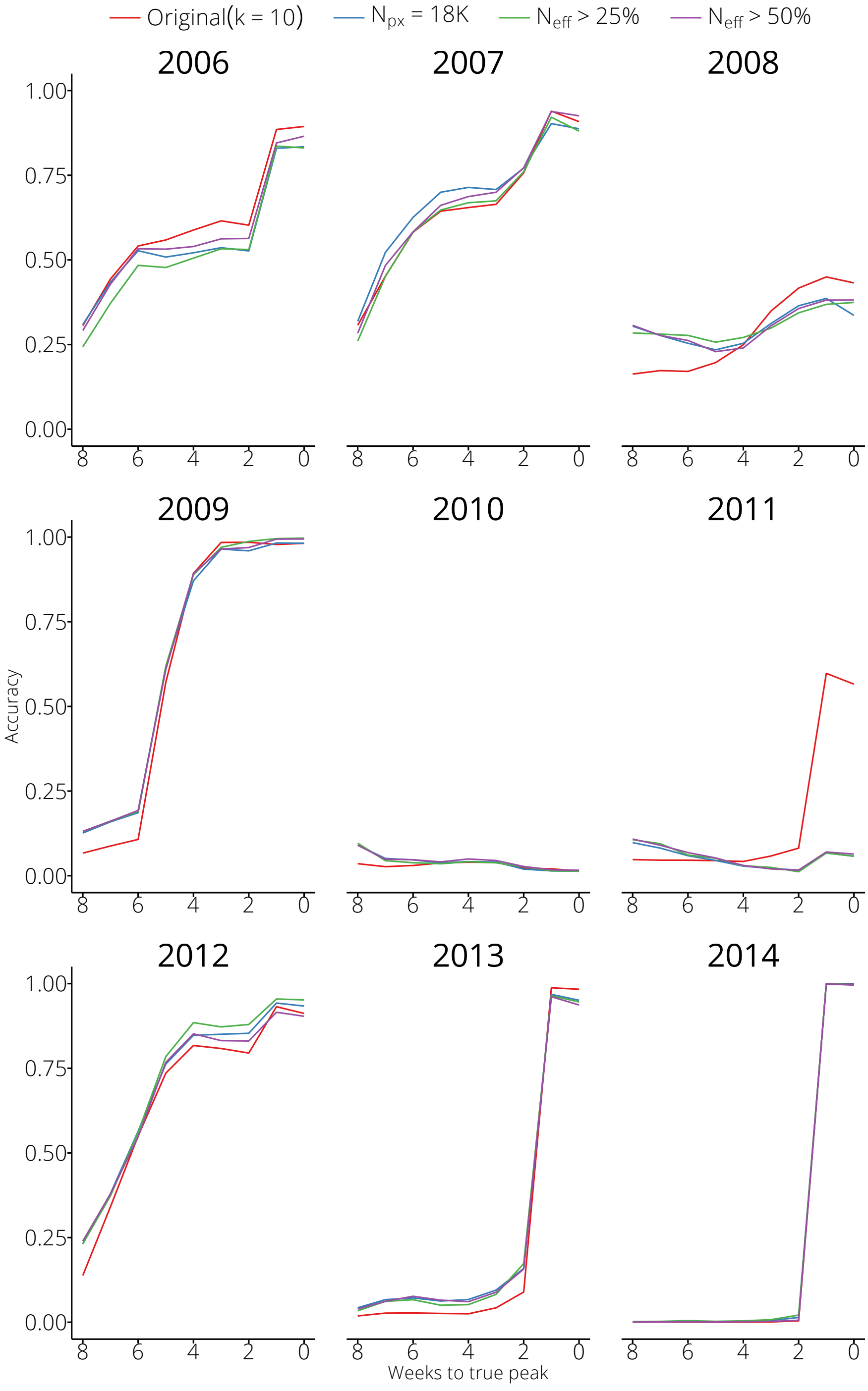

Figure S18: A comparison of our original forecasts ($k = 10$) with forecasts generated using the original model where the number of particles was increased five-fold ("$N_{px} = 18K$") and where the resampling threshold was decreased ("$N_{\text{eff}} > 25\%$" and "$N_{\text{eff}} > 50\%$").

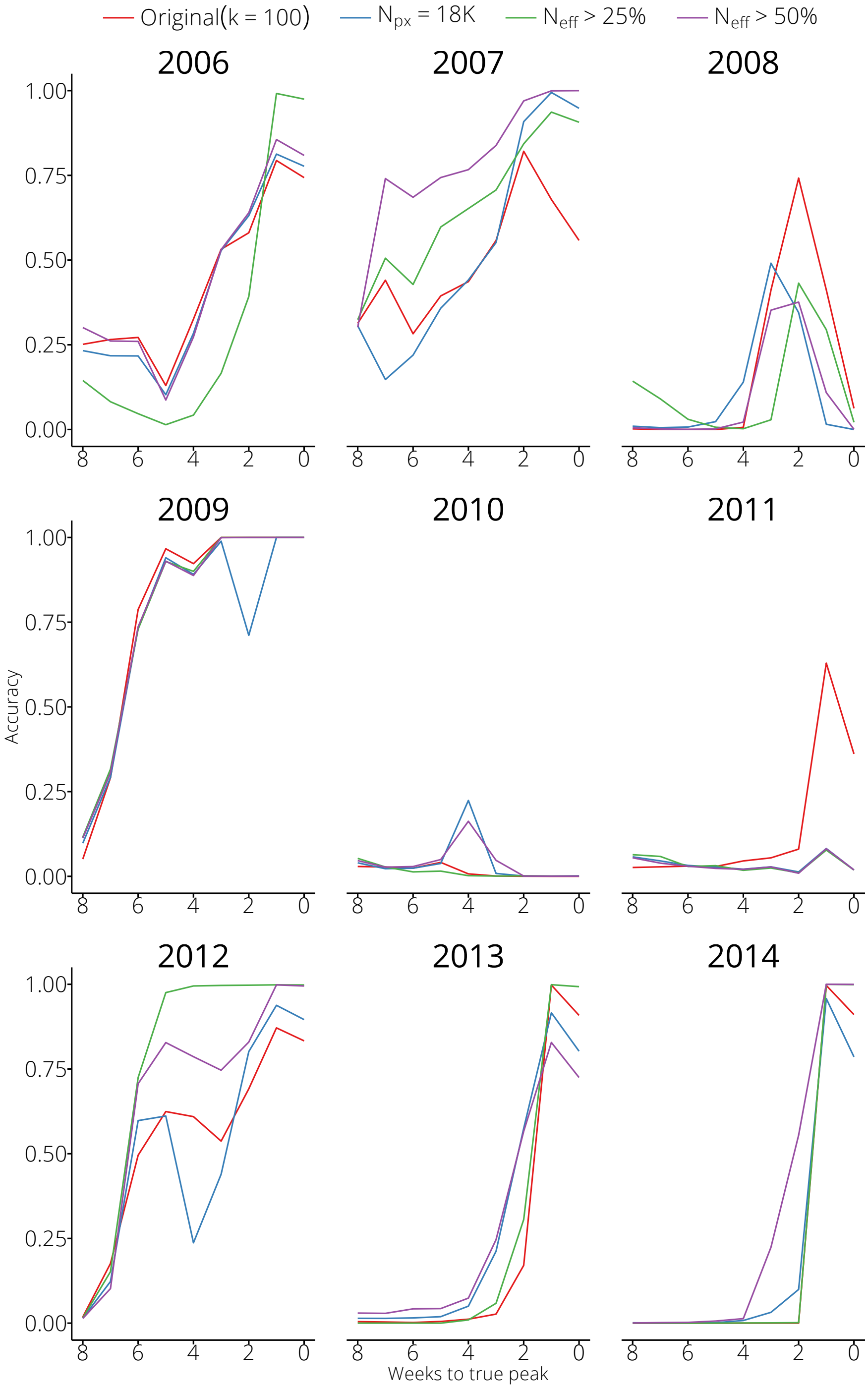

Figure S19: A comparison of our original forecasts ($k = 100$) with forecasts generated using the original model where the number of particles was increased five-fold ("$N_{px} = 18K$") and where the resampling threshold was decreased ("$N_{\text{eff}} > 25\%$" and "$N_{\text{eff}} > 50\%$").

S6 References

- Kitagawa G. Monte Carlo filter and smoother for non-Gaussian nonlinear state space models. J Comp Graph Stat. 1996;5(1):1–25. Available from: http://www.416tandfonline.com/doi/abs/10.1080/10618600.1996.10474692.

- Skvortsov A, Ristic B. Monitoring and prediction of an epidemic outbreak using syndromic observations. Mathematical Biosciences. 2012 Nov;240(1):12–19. Available from: http://dx.doi.org/10.1016/j.mbs.2012.05.010.

- Department of Health & Human Services. 2013 Local government area profiles. Victorian Government; 2013. Accessed 2014-09-03. Available from: http://www.health.vic.gov.au/modelling/planning/lga.htm.

- Shaman J, Karspeck A. Forecasting seasonal outbreaks of influenza. Proc Natl Acad Sci USA. 2012 Dec;109(50):20425–20430. Available from: http://dx.doi.org/10.1073/pnas.1208772109.
